# Pre-adsorption of serum albumin on biomaterial surfaces modulates bacteria-surface interactions and alters bacterial physiological responses

**DOI:** 10.1016/j.mtbio.2025.102254

**Published:** 2025-08-30

**Authors:** Hung Le, Marie Droniou, Lisa Wallart, Laurent Coquet, Pascal Thebault, Clément Guillou, Pascal Cosette

**Affiliations:** a Univ Rouen Normandie, INSA Rouen Normandie, CNRS, Normandie Univ, PBS UMR 6270, Rouen, France; b Univ Rouen Normandy, INSERM US 51, CNRS UAR 2026, HeRacLeS PISSARO, Rouen, France

**Keywords:** Bacteria-surface interactions, Serum albumin adsorption, *Pseudomonas aeruginosa*, Bacterial adhesion, Biofilm, Proteome

## Abstract

When a biomedical device is implanted into the body, its surface initially encounters biological fluids, resulting in the natural adsorption of various host proteins. This protein-adsorbed layer alters the inherent properties of the biomaterial surface and plays a crucial role in interactions between the implant and bacteria. Here, we investigated the influence of an adsorbed layer of albumin, the most abundant blood protein, on the adhesion and biofilm formation of three different bacterial strains: *Staphylococcus aureus*, *Staphylococcus epidermidis*, and *Pseudomonas aeruginosa*. We found that the effect of a serum albumin layer on bacterial adhesion was strain-dependent. Albumin pre-adsorption reduced the initial attachment of *S. aureus* and *S. epidermidis* to PDMS surfaces but had no impact on *P. aeruginosa*. However, with prolonged incubation, albumin-coated surfaces significantly promoted *P. aeruginosa* attachment and biofilm formation. Additionally, this biofilm alteration was associated with reduced macrophage-mediated bacterial clearance. Proteomic analysis further revealed significant physiological changes in *P. aeruginosa* upon exposure to albumin-coated surfaces compared to uncoated controls. These alterations were particularly associated with molecular pathways involved in surface colonization, including *quorum sensing*, motility, adhesion, and biofilm formation. These findings suggest that serum albumin adsorption not only affects the initial adhesion of bacteria but also modifies their adaptive responses upon contact with the biomaterial surface. This study provides a deeper understanding of the complex bacteria-surface interactions, contributing to the development of future strategies for preventing implant-associated infections.

## Graphical abstract

**Figure:**
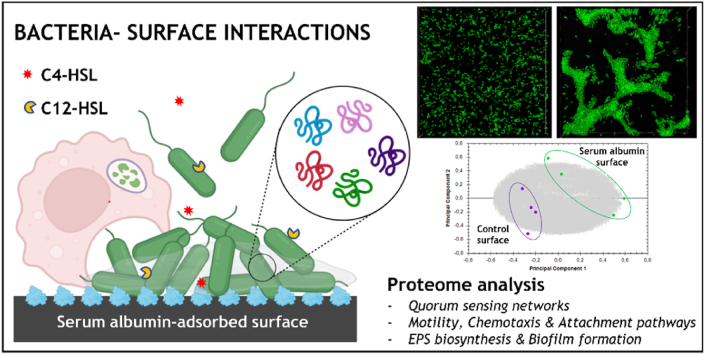

## 1 Introduction

Biomaterials play an increasingly vital role in the success of tissue engineering and indwelling medical devices, significantly enhancing patients’ quality of life. However, infections related to biomaterials present significant challenges that restrict their application [1]. These infections may occur from contamination during surgery or postoperative haematogenous spread from other infection sites within the body. After adhesion on biomedical devices, bacteria frequently tend to produce a matrix of extracellular polymeric substances (EPS) composed of polysaccharides, proteins, eDNA, and lipids [2]. This self-generated matrix encases the bacteria, forming a robust biofilm that shields them from antibiotics and the host immune system. Treating these infections is challenging and often leads to serious complications, with surgical removal of the infected device frequently being the only effective option [3].

Despite substantial efforts to develop antifouling or antibacterial materials to lower infection risks during implantation, current clinical observations suggest that these measures remain insufficient [4]. The challenges in developing infection-resistant materials can be attributed to several factors. First, the interactions between bacteria and surfaces are highly complex, involving physicochemical parameters from both the bacterial surface and the material surface. Additionally, when materials are introduced into the body, various host factors, such as components of biological fluids or immune cells, can also influence these interactions [5]. Moreover, bacteria are able to sense surfaces upon contact and can trigger a series of responses that ultimately lead to biofilm formation [2]. A complete understanding of material surface properties, the impact of environmental factors at the implant site, and bacterial physiology is therefore essential for developing effective methods to prevent these infections [6].

When studying biomaterial-associated infections, a critical yet often overlooked factor is protein adsorption on the implant surface [7]. This process is one of the first biological events that occurs when a foreign material is introduced into the body, far before the adhesion and colonization of bacteria. For example, adsorbed albumin has been identified on almost all types of surfaces in contact with blood serum or plasma [8]. Similarly, during urinary catheterization, the host's inflammatory response leads to the deposition of proteins like factor C3, fibrinogen, and serum albumin (SA) onto the catheter [9]. This phenomenon leads to protein-coated biomaterial surfaces acquiring physicochemical properties distinct from those of the underlying material, thereby influencing the interactions between the biomaterial and bacteria [10]. A prime example is the effect of SA, the most abundant protein in plasma, on the surface of biomaterials. Some studies suggest that pre-adsorption of SA can prevent subsequent non-specific protein adsorption and bacterial adhesion, while others report that an SA layer may enhance bacterial adhesion and biofilm formation [[9], [10], [11], [12]]. Despite these conflicting findings, several studies have proposed pre-coating biomaterial surfaces with SA as a strategy to improve biocompatibility and reduce the risk of infection [8]. However, a recent clinical study found no significant antibacterial effects of albumin-coated surfaces [13].

Given the complexity of bacteria-surface interactions, we hypothesize that, beyond the physicochemical changes induced by protein adsorption, bacteria may actively sense the presence of these host proteins and adapt their behavior on the modified surfaces. To test this hypothesis, we investigate whether the SA-pre-adsorbed layer influences bacterial adaptive responses on biomaterial surfaces.

In this study, polydimethyl siloxane (PDMS) surface, a widely used material in biomedical applications, was synthesized and used as a substrate for protein adsorption experiments. The physicochemical characterization of these surfaces was first performed to confirm the presence of the albumin layer. Then, the impact of the albumin layer on the adhesion and biofilm formation was examined with three major species commonly associated with infections of medical devices: *S. aureus*, *S. epidermidis*, and *P. aeruginosa.* Based on the observed phenotypic outcomes, further investigations specifically focused on the biofilm characteristics of *P. aeruginosa* formed on SA-adsorbed surfaces. Lastly, the proteome and *quorum-sensing* signal molecules of adhered *P. aeruginosa* were analyzed using mass spectrometry to elucidate the bacterial adaptation to the presence of a SA layer compared to unmodified surfaces. To the best of our knowledge, this is the first study to describe bacterial adaptation processes on SA-preadsorbed surfaces using a proteomic approach.

## 2 Material and methods

### 2.1 Bacterial strains and growth conditions

Microbiological experiments were conducted using three standard laboratory bacterial strains: *S. aureus* ATCC 12600, *S. epidermidis* ATCC 35984, and *P. aeruginosa* PAO1. Bacteria from a frozen stock at −80 °C were first grown overnight at 37 °C on Mueller Hinton (MH) (Difco™) agar plates. A single colony was then used to inoculate 5 mL of MH Broth (Difco™) and grown overnight (16 h, 37 °C, 147 rpm shaking) before all experiments.

### 2.2 Preparation of polydimethyl siloxane surfaces

Polydimethyl siloxane (PDMS) surfaces were prepared using Sylgard® 184 with a silicone elastomer base to curing agent ratio of 10:1 (w/w). Briefly, 15 g of the mixture was poured into a flat petri dish, degassed under vacuum to remove air bubbles, and cured at 70 °C for 3 h. The resulting PDMS film (with a thickness of 1 mm) was cut into 1 cm^2^ square surfaces using a scalpel. These PDMS surfaces were sterilized under UV light for 30 min and stored at room temperature.

### 2.3 Albumin pre-adsorption and surface characterization

Lyophilized serum albumin (SA) (A9418, Sigma Aldrich) powder was initially dissolved in phosphate-buffered saline (PBS) at physiological concentrations of 50 mg/mL and 5 mg/mL. Protein adsorption was achieved by submerging the PDMS surfaces in the SA solutions for 24 h at 37 °C. Control surfaces were prepared in the same manner but without SA. After incubation, the surfaces were removed and rinsed three times with Milli-Q water to remove any non-adhered protein. The SA-adsorbed substrates were then dried with an argon stream and either used immediately for bacterial adhesion experiments or stored at 4 °C.

Concerning the surface characterization, the FTIR spectra were recorded on a Nicolet IS-50 FTIR spectrometer (Thermo Scientific) using the Smart™ iTX accessory (Thermo Scientific) and the DTGS detector. Spectra were collected from three independent samples for each experimental condition, covering a wave-number range of 500 to 4000 cm^−1^. Spectra were obtained with a spectral resolution of 2 cm^−1^ and averaged from 64 scans.

The hydrophilicity of the surface was evaluated by measuring the water contact angle (WCA) formed between water droplets and the surfaces using a goniometer (Krüss). Briefly, a 10 μL drop of Milli-Q water was deposited on the surface at room temperature with a microsyringe and a measure was immediately performed using the ADVANCE software supplied with the device. Measurements were conducted on at least three independent samples, with a minimum of three droplets per sample placed at different locations on each surface.

Additionally, the distribution of adsorbed SA on PDMS surfaces was observed using confocal laser scanning microscopy (CLSM). The protein adsorption protocol was followed, utilizing fluorescent FITC-labeled SA (A23015, Thermo Fisher Scientific). A 2 μL drop of Milli-Q water was then placed on the PDMS surfaces, followed by a round glass slide with a diameter of 0.8 cm. The surfaces were observed using a Leica TCS SP8 confocal laser-scanning microscope (Leica Microsystems, Germany). FITC-labeled SA was excited at 488 nm, and their fluorescence emissions were collected through HyD detectors (counting mode) between 500 and 540 nm. Image processing was performed with Imaris (Bitplane, Switzerland) software. The amount of SA adsorbed onto the PDMS surface was estimated using a gradient concentration of FITC-labeled SA on PDMS surfaces. Specifically, 2 μL aliquots of FITC-labeled SA at concentrations ranging from 1 to 50 mg/mL were deposited onto untreated PDMS surfaces. A round glass slide with a diameter of 0.8 cm was then placed on top, creating surfaces with protein concentrations from 2 to 50 μg/cm^2^. Fluorescence image acquisition was performed as described above, and signal intensity was measured consistently in the same area for each sample. The correlation between fluorescent intensity and the amount of FITC-labeled SA (μg/cm^2^) was established using the equation y = 423427x + 982661 (R^2^ = 0.9841).

### 2.4 Bacterial adhesion test and biofilm formation study

The overnight culture of bacteria was centrifuged and washed once with PBS. The bacterial pellets were then resuspended in fresh MH media at a concentration of 2 × 10^6^ CFU/mL. One mL of this bacterial suspension was added to 24-well plates containing either untreated or SA-pre-adsorbed PDMS surfaces. The plates were incubated under static conditions at 37 °C for 2 h to test adhesion or 24 h to test biofilm formation. After incubation, the surfaces were rinsed three times with PBS to remove non-adhered bacteria and then transferred to Falcon tubes containing 5 mL of PBS. The tubes were sonicated for 10 min at 45 kHz to detach bacterial cells attached to the surfaces. The bacterial suspensions were then plated for enumeration using the microdilution method. After 24 h of incubation at 37 °C, colonies were counted and expressed as CFU/cm^2^. Each assay was performed in triplicate with three independent enumerations.

Adhered bacteria on PDMS substrates were also visualized using CLSM. The bacterial cells were stained with either 10 μM hexidium iodide or 20 μM SYTO 9 (both from Thermo Fisher Scientific) for 15 min at room temperature before visualization. The PDMS substrates were mounted onto a glass microscope slide using a Mowiol 4-88 mounting medium. Images were acquired using a Leica TCS SP8 CFS confocal microscope with a fixed stage (Leica Microsystems), equipped with a 488 nm diode laser (Coherent) for excitation. Fluorescence emission was detected sequentially using a hybrid detector (Leica Microsystems) in photon counting mode, with a specific band from 500 to 540 nm for SYTO 9 and 570-610 for hexidium iodide. Image processing was carried out using Imaris software (Bitplane).

### 2.5 Quantification of biomass and bacterial density of biofilm

Cell density within the biofilm was determined using CLSM. Biofilms were stained with SYTO 9 for 30 min following the manufacturer's protocol before microscopy. The surface was then mounted onto a glass microscope slide using a Mowiol 4-88 mounting medium. Image acquisition and processing were performed as detailed in Section 2.5. Imaris software (Bitplane) was used to quantify the biofilm's biovolume (μm^3^/μm^2^).

Biomass quantification of *P. aeruginosa* biofilm on different surfaces was conducted using crystal violet (CV) staining. In brief, biofilms on PDMS substrates were washed twice with PBS and then stained with 1 mL of 0.1 % CV for 15 min. After removing the CV, the wells were rinsed twice with 1 mL PBS. The CV bound to the biomass was solubilized with 1 mL of 30 % acetic acid. The wells were homogenized, and the CV solution was transferred to a new plate for optical density measurement at 580 nm using a microplate reader (FlexStation 3, Molecular Devices, UK). All assays were performed in triplicate across at least three independent experiments.

### 2.6 Macrophage-mediated killing assay

Biofilms of *P. aeruginosa* were formed on PDMS surfaces, either unmodified (Control) or pre-adsorbed with serum albumin (SA50), as described in Section 2.5. This experiment used a PAO1 strain expressing enhanced green fluorescent protein (eGFP) to facilitate microscopic observation [14]. The eGFP coding sequence was placed under the control of the PA4249 gene promoter, which is constitutively expressed in both planktonic and sessile PAO1 cells. After 24 h of incubation, the surfaces were gently rinsed twice with PBS and transferred to fresh 24-well plates for the macrophage-mediated killing assay.

Murine RAW 264.7 macrophages were cultured as monolayers in Dulbecco's Modified Eagle Medium (DMEM; Gibco™, Thermo Fisher Scientific), supplemented with 10 % heat-inactivated fetal bovine serum and antibiotics (100 U/mL penicillin G and 100 μg/mL streptomycin; Thermo Fisher Scientific). Cells were maintained at 37 °C in a humidified atmosphere containing 5 % CO_2_. On the day of the experiment, RAW 264.7 monolayers were washed twice with PBS to remove serum prior to trypsinization. The detached cells were then washed again and resuspended in fresh medium without antibiotics. A volume of 1 mL of this suspension containing 7 × 10^5^ cells/mL was seeded into 24-well plates containing biofilms preformed on PDMS surfaces.

After 1 h incubation, the surfaces were rinsed three times with PBS and transferred to Falcon tubes containing 5 mL of PBS. The tubes were sonicated at 45 kHz for 10 min to detach bacteria from the surfaces. The resulting bacterial suspensions were plated and enumerated using the microdilution method. For microscopy experiments, following the 1-h incubation, the surfaces were rinsed twice with PBS, fixed with 4 % formaldehyde for 20 min, and permeabilized with 0.1 % Triton X-100 for 10 min. Samples were then stained in the dark for 30 min with a mixture of Rhodamine Phalloidin ActinRed™ 555 (for F-actin visualization) and DAPI (for nuclear staining), followed by two additional PBS washes. Finally, the samples were mounted onto glass microscope slides using Mowiol 4-88 mounting medium (Sigma-Aldrich). Image acquisition was carried out using a Leica TCS SP8 CFS confocal microscope. DAPI, eGFP, and ActinRed were excited at 405, 488, and 552 nm, respectively. Fluorescence emission was collected sequentially using a hybrid detector in photon-counting mode, with band-pass filters set at 450–480 nm for DAPI, 500–540 nm for eGFP, and 570–620 nm for ActinRed. Image analysis and cell quantification were performed using Imaris 9.8 software, with the “spot fluorescent detection” function used to automatically determine cellular density.

### 2.7 Proteomics analysis of adhered bacteria on the surfaces

#### 2.7.1 Protein extraction

To obtain sufficient biomass for proteomic analysis, the bacteria were cultured on 1 g of glass wool (10 μm diameter) as described previously [14]. The concentration of 24 h-adhered bacteria on glass wool, with or without pre-adsorbed SA, was assessed through agar enumeration and CLSM under the same experimental conditions mentioned above. Each growth condition was performed in four biological replicates. Intracellular protein extractions from the bacteria adhered to the surfaces were performed as previously described [15]. After rinsing the glass wool with PBS (3 × 10 mL) to remove planktonic cells, bacteria were recovered by adding sterilized glass beads (1 g in 10 mL of PBS) and agitating for 5 min. This step was repeated three times to maximize the recovery of adhered bacteria. The recovered cells were then washed three times with PBS (3 × 10 mL) using centrifugation at 3000×*g* for 15 min to remove extracellular proteins. The final pellet was resuspended in extraction buffer (7 M urea, 2 M thiourea, 4 % 3-[(3-cholamidopropyl) dimethylammonio]-1-propanesulfonate hydrate (CHAPS), 65 mM dithiothreitol (DTT) and 25 mM Tris/HCl), supplemented with a protease inhibitor cocktail (10 μL/mL, Protease Inhibitor Cocktail-Bacterial, Sigma-Aldrich) to prevent protein degradation. The mixture was freeze-thawed for three cycles and then sonicated on ice six times, each for 1 min. Finally, the lysate was centrifuged at 9000×*g* for 25 min at 4 °C to recover the supernatant containing the intracellular proteins. The protein concentrations were determined using the Bradford assay (BioRad). Additionally, SDS-PAGE was carried out on the protein extracts to check the consistency of the protein profiles. Samples were stored in aliquots at −20 °C until further use.

#### 2.7.2 Enzymatic digestion

Twenty-five micrograms of proteins were mixed with a loading buffer (63 mM Tris-HCl, pH 6.8, 10 mM DTT, 2 % SDS, 0.02 % bromphenol blue, 10 % glycerol) and loaded onto a 7 % SDS-PAGE stacking gel. Electrophoresis was conducted at 10 mA for 105 min to concentrate the proteins. After migration and staining, the visualized protein band was excised, washed three times with water, and incubated in a reductive buffer (5 mM DTT). Cysteines were alkylated with 25 mM iodoacetamide for 30 min in the dark. Following washing steps using water, gel bands were treated with 50 % acetonitrile (ACN)/50 % ammonium bicarbonate 10 mM, pH 8 (2 times, 5 min) and dried with 100 % ACN (3 times, 10 min). Proteins were digested with trypsin (1:25 mass ratio) overnight at 37 °C in ammonium bicarbonate (10 mM, pH 8). Peptides were extracted using 3 ACN: H_2_O baths and dried.

#### 2.7.3 NanoLC-MS/MS protein analyses

After the digestion of proteins, peptide fractions were solubilized in 0.1 % formic acid (FA) (v/v) and analyzed using mass spectrometry. All the experiments were performed using Q-Exactive Plus equipment (Thermo Scientific, Waltham, MA, United States) coupled with an Easy-nLC 1200 (Thermo Scientific). One μL of sample (0.2 μg) was injected and first eluted toward an enrichment column (C18 Pepmap100 precolumn [300 μm ID × 5 mm, 5 μm, 100 Å, Thermo Scientific]). The separation was carried out using a PepMap RSLC C18 (Thermo Scientific). The mobile phases are composed of 100 % H_2_O/0.1 % FA (A) and 80 % ACN/20 % H_2_O/0.1 % FA (B). The tryptic peptides were eluted at a flow rate of 300 nL/min for 120 min using a three-step linear gradient: 2 % to 35 % B from 0 to 84 min, 35 % to 90 % B from 84 to 94 min, 90 % B from 94 to 99 min, and 2 % B from 99 to 120 min. The mass spectrometer was operated in the positive ionization mode with the capillary voltage and the source temperature set at 1.6 kV and 275 °C, respectively. The first scan (MS spectra) was recorded using the Orbitrap analyzer (R = 70,000) in the mass range of *m/z* 400–1800. Automated Gain Control target and maximum injection time were set to 3 × 10^6^ and 60 ms, respectively. Then, the 10 most intense ions (Top 10) were selected for tandem mass spectrometry (MS2) experiments, excluding monocharged ions. The dynamic exclusion of the already fragmented precursor ions was carried out for 20 s. Fragmentation occurred in the HCD cell analyzer at a normalized collision energy of 28 V. The MS2 spectra were also recorded using the Orbitrap analyzer at a lower resolution (R = 17,500).

#### 2.7.4 Protein identification and quantification

A label-free experiment was performed as previously described [15]. Briefly, the raw data were imported into the Progenesis LC-MS software (Nonlinear Dynamics, version 4.1, Newcastle, UK). A 2-dimensional map was generated for each sample (retention time *versus m/z* ratio). For the differential analysis, one sample was set as a reference, and the retention times of all the other samples within the experiment were aligned. Monocharged ions and those with a charge greater than 5 were excluded from the analysis. After the alignment and normalization, the MS/MS spectra of the selected peptides were exported for peptide identification with Mascot (Matrix Science, version 2.2.04) against the *P. aeruginosa* PAO1 database available online (https://www.pseudomonas.com/downloads/sequences). Database searches were performed with the following parameters: 1 missed cleavage site, and two variable modifications (carbamidomethylation of cysteine and oxidation of methionine). The mass tolerances for the precursor and fragment ions were set at 5 ppm and 0.02 Da, respectively. False discovery rates (FDR) were calculated using a decoy-fusion approach in Mascot (version 2.2.04) and set to 1 %. Then, Mascot search results were re-imported into Progenesis software. Proteins identified with less than 2 peptides were discarded. For each growth condition, the total cumulative abundance of each protein was calculated by summing the abundances of peptides. The abundances were normalized to perform relative quantification of each protein within the 2 conditions. To highlight the proteins exhibiting a differentially significant expression, the following statistical filters were applied at the protein level: p-value <0.05 and q-value <0.05. The MS proteomics data have been deposited in the ProteomeXchange Consortium *via* the PRIDE partner repository with the data set identifier PXD058993.

### 2.8 Quantification of quorum sensing by HPLC-MS/MS

The biomolecules involved in the *quorum sensing* of *P. aeruginosa* were quantified in the surrounding media of *P. aeruginosa* biofilm, following a protocol previously described [16]. 24 h-*P. aeruginosa* cultures, in the presence of unmodified or SA-adsorbed surfaces, were centrifuged, and the supernatants were collected and filtered to eliminate the planktonic cells. The supernatants were then extracted twice with equal volumes of acidified ethyl acetate (0.01 % v/v glacial acetic acid in ethyl acetate). The organic layers were collected, evaporated, and stored at −20 °C.

The quantification of N-butanoyl-homoserine lactone (C4-HSL) and N-(3-oxododecanoyl)-L-homoserine lactone (3-oxo-C12-HSL) was carried out using HPLC-MS/MS. First, the organic fraction extracts were resuspended in a solution of 50 % ACN and 0.1 % FA in water, sonicated twice for 15 min, and then centrifuged for 10 min at 7000×*g*. N-heptanoyl-L-homoserine lactone (C7-HSL) was added to all samples at a final concentration of 250 pg/μL as an external standard since this homoserine lactone is not naturally produced by *P. aeruginosa*.

Separation of the biomolecules was performed with an Agilent 1290 Infinity II HPLC system coupled to a 6545XT AdvanceBio Q-TOF mass spectrometer (Agilent Technologies, USA). The column used was a Zorbax Eclipse Plus C18, 2.1 mm × 100 mm with 1.8 μm particle size (Agilent Technologies, USA). The mobile phase buffers were 0.1 % FA in water (A) and 90 % ACN with 0.1 % FA in water (B). The constant flow rate was 0.4 mL/min, and the column temperature was set at 60 °C. The elution gradient lasted 18 min as follows: 3 % to 10 % B for 3 min, 10 % to 45 % B for 1 min, 45 % to 75 % B for 1 min, 75 % to 90 % B for 3.5 min, a flushing phase at 90 % B for 4 min and a re-equilibration phase at 3 % B for 5.5 min. One microliter of diluted samples was injected into the column. The mass spectrometer operated in positive electrospray ionization mode and full scan. The settings were as follows: Drying Gas, 12 L/min; Nebulizer, 60 psi; Sheath Gas Temperature, 200 °C; Sheath Gas Flow, 11 L/min; Capillary Voltage, 3500 V; Skimmer, 65 V. The ESI source gas temperature and the MS-TOF fragmentor voltage were set at 150 °C and 100 V, respectively, to enhance biomolecule detection sensitivity. Moreover, the “fragile ions” mode was also activated. The *m/z* value was scanned from 50 to 950 in both MS and MS/MS modes, with data collected at 2 spectra/s and a maximum of 5 precursors for fragmentation in MS/MS, with collision energies of 5, 10, 15, 20, 25, and 30 V.

The MS data analysis was performed using Agilent MassHunter software (version B.07.00). C4-HSL and 3-oxo-C12-HSL were detected at [M + H^+^] *m/z* values of 172.09 and 298.20, respectively, with a tolerance of ± 0.02 Da. Identification was confirmed by specific ion fragments, particularly the lactone-ring ion (*m/z* = 102.055) for the AHL molecules. The area of each EIC peak, corresponding to the relative abundance of [M + H^+^] ions detected in the MS spectra, was measured (see Fig. S1). Only areas with values that fell within the linear calibration curves established with standard molecules were retained for quantification. The amount of AHLs in the samples was determined by comparing the ratio of [C4-HSL or 3-oxo-C12-HSL/C7-HSL peak area] to a calibration curve.

### 2.9 Statistical analysis

All data are presented as mean ± standard error of the mean (SEM). Statistical analyses were performed using GraphPad Prism software, version 7.0. The statistical differences between the groups were determined using a non-parametric test (Mann-Whitney). Significance values on graphs are ∗p ≤ 0.05, ∗∗p ≤ 0.01, ∗∗∗p ≤ 0.001, and ∗∗∗∗p ≤ 0.0001.

## 3 Results and discussion

### 3.1 Validation of serum albumin pre-adsorption on the PDMS surface

**Fig. 1:**
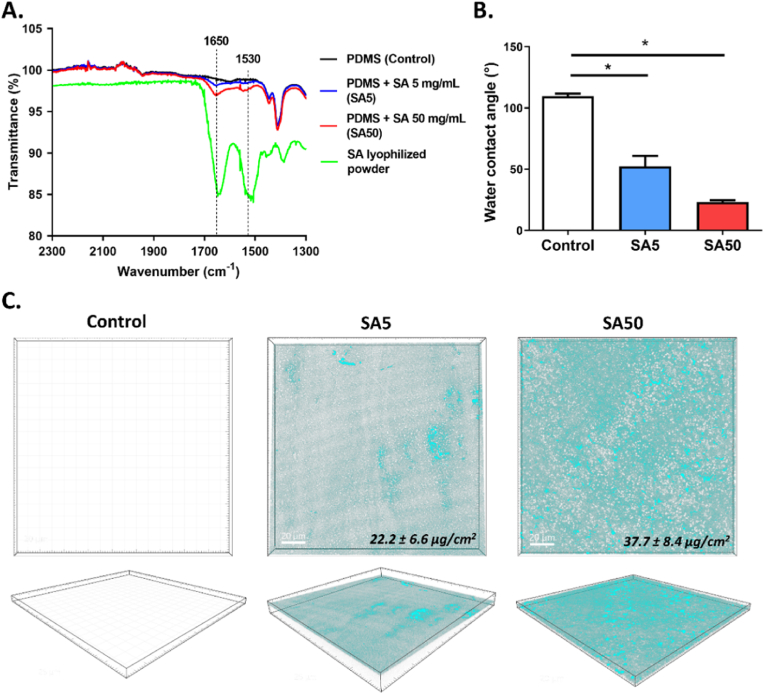
**Validation of serum albumin adsorption on PDMS surface. (A**) FTIR spectra of the unmodified PDMS surface (Control), PDMS surfaces absorbed with SA at concentrations of 5 mg/mL (SA5) and 50 mg/mL (SA50), and pure SA lyophilized powder. (**B**) Water contact angle of Control, SA5 and SA50 surfaces. (**C**) 3D Representative images of adsorbed SA on the PDMS surface after incubation with fluorescent SA-FITC conjugate at concentrations of 5 mg/mL (SA5) and 50 mg/mL (SA50). Quantification of SA adsorbed on the PDMS surface was conducted using a range of SA-FITC concentrations from 0 to 40 μg/cm^2^. Data are represented as mean ± SEM; n = 3, ns: not significant; ∗p < 0.05.

The present study aims to evaluate the effect of serum albumin adsorption on the interaction between bacteria and the surface of a biomaterial. Polydimethyl siloxane (PDMS) surface was used as a control substrate due to its widespread use in biomedical applications, especially in indwelling medical implants [17]. Two SA concentrations, 5 mg/ml and 50 mg/ml (designated as SA5 and SA50 conditions), were used to simulate albumin levels in biological fluids when in contact with material surfaces [18]. After 24 h of incubation, the PDMS surface was washed thrice with Milli-Q water and dehydrated before experiments. As shown in Fig. 1A and S2, the FTIR spectrum of PDMS with adsorbed SA on the surface exhibits two new peaks at 1650 cm^−1^ and 1530 cm^−1^. Compared to the FTIR spectrum of pure SA powder, these peaks correspond to the amide I (C=O stretching and C-N stretching vibration) and amide II bands (NH in-plane bending vibration and C-N stretching vibration) of the peptide bonds of SA, respectively, with a shift of about 3 cm^−1^. This slight shift in the amide peak characteristics between adsorbed SA and SA powder could be attributed to conformational changes occurring after interaction with the surface [18,19].

Additionally, the SA adsorption altered the non-polar character of the PDMS surface, reducing the water contact angle (WCA) value from 110° to 49° and 23° after incubation with 5 mg/mL and 50 mg/mL SA, respectively (Fig. 1B). The reduction of the WCA values after SA contact suggested that the PDMS surfaces became more hydrophilic in the presence of SA layer, with the effect becoming more pronounced at higher SA concentration. These results align with previous studies demonstrating that a thin protein layer could enhance surface hydrophilicity and help prevent adverse biological reactions when materials come into contact with blood [20]. Based on this point, biomaterial surfaces could be incubated in an SA solution before implantation to enhance biocompatibility, a process known as "albumin passivation" [8]. For example, in several preclinical and clinical studies, albumin-coated materials have been shown to reduce the number of adherent platelets and their activation on the surface [11,13,[21], [22], [23]].

The presence and coverage of the adsorbed protein layer were further verified through fluorescence microscopy using FITC-labeled SA. As illustrated in Fig. 1C, PDMS substrates that did not undergo the protein incubation step did not exhibit autofluorescence. After 24 h of SA incubation, a continuous layer of SA formed on the PDMS surface in both conditions. However, SA adsorbed from the higher concentration solution was shown to be more uniformly distributed and thicker. These findings align with previous studies indicating that SA adsorption is a concentration- and time-dependent kinetic process [24]. The amount of SA adsorbed on the surfaces was quantified by measuring the fluorescent signal on the sample surfaces. The adsorption of SA increased with its concentration in the medium, reaching about 22 μg/cm^2^ for SA5 and 38 μg/cm^2^ for SA50. These values are slightly higher than those reported in some previous studies, which indicated SA adsorption onto biomaterial surfaces in the range of 10–30 μg/cm^2^ [25]. This discrepancy may result from differences in the physicochemical properties of the tested surfaces, incubation conditions for protein adsorption, or the methods used to quantify SA. For instance, most previous studies used indirect measurements to assess the amount of adsorbed protein on surfaces, typically involving a detachment step followed by quantification of the free protein in the medium. A limitation of this approach is that incomplete detachment of proteins, influenced by the strength of protein-surface interactions, can lead to an underestimation of the adsorbed proteins [19]. In our study, the concentration of SA was directly measured on the surface, which may explain the slightly higher SA levels compared to those reported in the literature.

### 3.2 Pre-adsorption of serum albumin on PDMS affects the adhesion of S. aureus, S. epidermidis, and P. aeruginosa

**Fig. 2:**
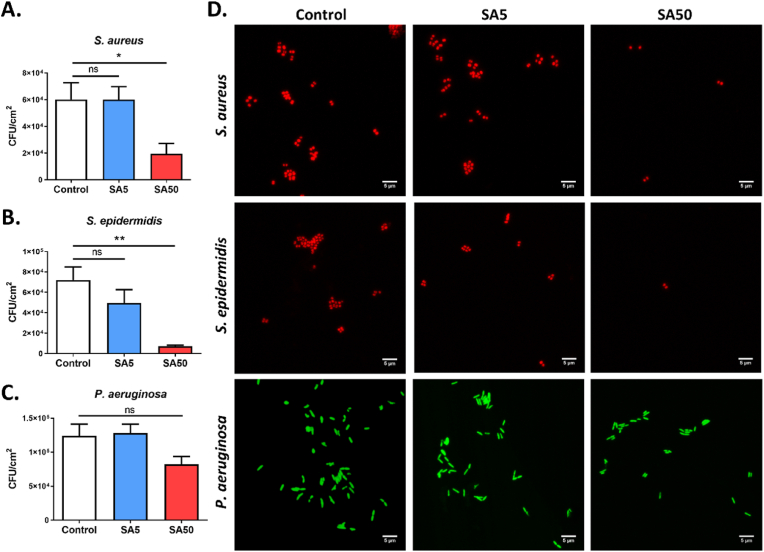
**Effect of pre-adsorption of serum albumin on the initial adhesion of *S. aureus*, *S. epidermidis* and *P. aeruginosa*.** Quantification of adhered *S. aureus***(A)**, *S. epidermidis***(B)**, and *P. aeruginosa***(C)** on the unmodified PDMS surface (Control) and the SA-pre-adsorbed PDMS surfaces (SA5 and SA50) after 2 h of incubation. **(D)** Representative confocal images showing bacterial adhesion on unmodified PDMS surfaces (Control) and SA-pre-adsorbed surfaces (SA5 and SA50) after 2 h of incubation. Gram-positive bacteria *S. aureus* and *S. epidermidis* were stained with hexidium iodide (488/600 nm), while the Gram-negative *P. aeruginosa* was stained with SYTO 9 (488/520 nm). Data are represented as mean ± SEM; n = 4, ns: not significant; ∗p < 0.05; ∗∗p < 0.01; Scale bar: 5 μm.

After confirming the presence of a SA layer on the PDMS surface, we assessed the impact of these adsorbed protein layers (SA5 and SA50) on the adhesion of three bacterial strains: two Gram-positive strains, *S. aureus* and *S. epidermidis*, and one Gram-negative strain, *P. aeruginosa*. These three species are among the most common causes of device-associated infections [26]. *S. aureus* and *S. epidermidis* are more frequently associated with infections of central venous catheters, mechanical heart valves, and prosthetic joints, while *P. aeruginosa* is more commonly isolated from endotracheal tubes in ventilator-associated pneumonia and catheters in urinary tract infections. Bacterial adhesion on the PDMS surface was measured after a 2 h incubation period. This timeframe represents the early stage of bacterial adhesion, when reversible attachment transitions to irreversible binding [20]. As shown in Fig. 2A and B, the presence of the SA layer decreased the adhesion of *S. aureus* and *S. epidermidis*, with a particularly strong effect observed with the SA50 layer. Under this condition, the density of adhered *S. aureus* bacteria decreased from 6 × 10^4^ CFU/cm^2^ to approximately 2 × 10^4^ CFU/cm^2^ and *S. epidermidis* from 7 × 10^4^ CFU/cm^2^ to about 8 × 10^3^ CFU/cm^2^. Although not statistically significant (p = 0.0952), we observe a reduction in *P. aeruginosa* adhesion in the presence of the SA50 layer. This reduction is consistent with the findings previously described and may be due to the increased hydrophilicity of the PDMS surface by the SA layer [[27], [28], [29], [30], [31]]. It has been reported that hydrophilic materials typically exhibit greater resistance to bacterial adhesion than hydrophobic materials [29,30]. In our study, the presence of the SA layer reduced the WCA of the PDMS surface, making it more hydrophilic and less susceptible to bacterial adhesion. Additionally, it cannot be ruled out that the anti-adhesive effect of the albumin layer may be attributed to its acidic structure, which increases the surface's net negative charge, thereby enhancing the repulsion between the electric double layers of the organism and the surface [32].

The adhesion of three bacterial strains on the PDMS surface was also assessed using confocal microscopy (CLSM). As shown in Fig. 2D and Fig. S3, the presence of the SA50 layer significantly reduced the adhesion of *S. aureus* and *S. epidermidis*, which is consistent with the bacterial count results. However, no significant difference was observed in the adhesion of *P. aeruginosa* with or without the SA pre-adsorption layer. This suggests that SA pre-adsorption exhibits an anti-adhesive effect primarily on the two Gram-positive bacteria. To explore whether differences in bacterial surface charge could explain this selective effect, we measured the zeta potential of the three strains and SA in solution (Fig. S4). SA exhibited a nearly neutral zeta potential in water, suggesting minimal impact on the surface charge of PDMS upon adsorption. Although *S. epidermidis* had a more negative surface charge, *S. aureus* and *P. aeruginosa* showed similar values. These results suggest that the weaker anti-adhesive effect of the SA layer on *P. aeruginosa* cannot be explained by bacterial surface charge alone, but is more likely due to fundamental differences in membrane characteristics between Gram-negative and Gram-positive bacteria. To gain further insight into how the adsorbed SA layer modulates surface properties related to bacterial adhesion, additional techniques such as quartz crystal microbalance with dissipation monitoring (QCM-D) could be employed in future studies. Indeed, a recent study reported increased adhesion of Gram-negative bacteria on surfaces coated with an adsorbed SA layer [9]. Specifically, *P. aeruginosa* and *Acinetobacter baumannii* demonstrated approximately 14 % and 10 % higher adhesion, respectively, compared to uncoated controls after 2 h of incubation (p < 0.05). *Escherichia coli*, *Enterococcus faecalis*, and *Klebsiella pneumoniae* also showed a slight increase in adhesion, but it was not statistically significant (p > 0.05) [9]. These results suggest that protein adsorption can significantly influence initial bacterial adhesion, though this effect varies depending on the bacterial species.

### 3.3 Serum albumin pre-adsorption does not affect biofilm formation for S. aureus and S. epidermidis but enhances biofilm formation of P. aeruginosa

**Fig. 3:**
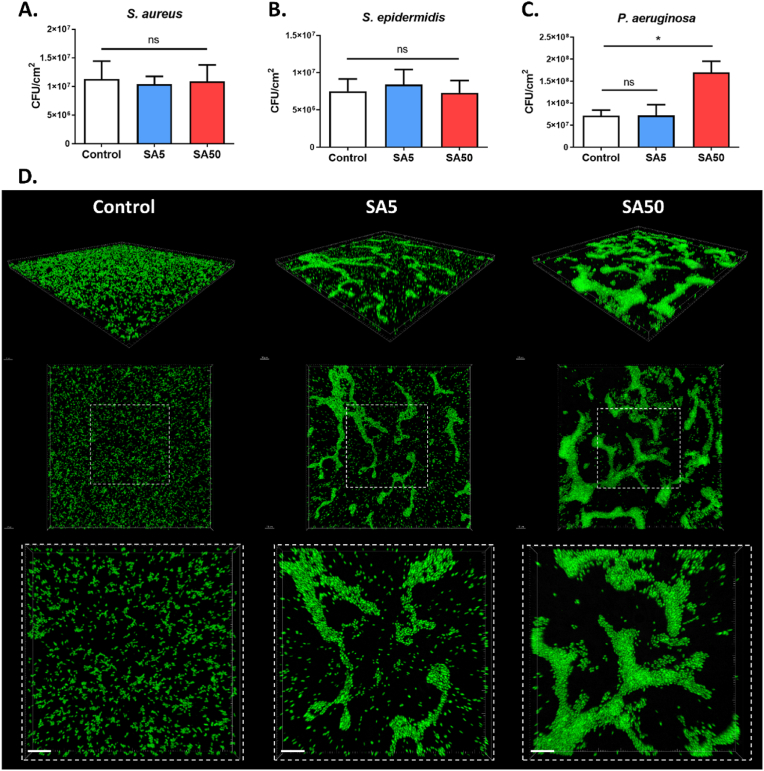
**Effect of serum albumin pre-adsorption on the bacterial biofilm formation.** Quantification of adhered *S. aureus* (**A**), *S. epidermidis* (**B**) and *P. aeruginosa* (**C**) on the unmodified PDMS surface (Control) and the SA-preadsorbed PDMS surfaces (SA5 and SA50); After 24 h of incubation with bacteria, the PDMS surfaces were rinsed three times with Milli-Q water and sonicated to detach the adhered bacteria. The suspended bacteria were then serially diluted and plated on MH agar for CFU enumeration and results are expressed as CFU/cm^2^. (**D**) Representative confocal images of biofilm formation of *P. aeruginosa* on unmodified PDMS surfaces (Control) and SA-pre-adsorbed surfaces (SA5 and SA50). Data are represented as mean ± SEM; n = 4, ns: not significant; ∗p < 0.05; Scale bar: 10 μm.

It is well known that the initial bacterial attachment can significantly impact subsequent biofilm formation and bacterial infection [26]. Therefore, we next evaluated whether the adsorbed SA layers on the PDMS surface could prevent bacterial adhesion at a longer interaction time (24 h). Longer interaction periods can facilitate biofilm maturation and the production of extracellular polymeric substances (EPS), thereby enhancing bacterial adhesion to surfaces. As shown in Fig. 3AB, for *S. aureus* and *S. epidermidis*, SA adsorption did not inhibit biofilm formation after 24 h of incubation, in contrast to the trend observed during the initial 2 h of contact. This suggests that the anti-adhesive effect of protein adsorption primarily occurs during the early stage of bacterial attachment. For longer incubation (24 h), the surface could suffer from the accumulation of bacteria and their exoproducts, leading to the obscuring of the SA layer and subsequent biofilm formation [20].

Although the SA layer did not significantly affect the initial adhesion of *P. aeruginosa*, an increase in the number of adhered bacteria on the SA50 pre-adsorbed surface (2.4 fold higher than uncoated controls, p < 0.05) was observed after 24 h of incubation (Fig. 3C). We questioned whether the increased bacterial adhesion might be related to a higher cell growth of *P. aeruginosa* in the presence of the SA layer. However, this hypothesis was ruled out because the presence of SA did not affect the concentration of *P. aeruginosa* in the surrounding environment (Fig. S5).

A confocal microscopy experiment and subsequent biovolume quantification of biofilm further confirmed these findings (Fig. S6). While no significant differences in bacterial density (μm^3^/μm^2^) were observed for *S. aureus* and *S. epidermidis* between SA-coated surfaces and the control, CLSM analysis of *P. aeruginosa* biofilms revealed an increased bacterial density on both SA5 and SA50 surfaces (Fig. S6C). Additionally, CLSM imaging revealed distinct morphological differences in *P. aeruginosa* biofilms between surfaces with and without the SA layer. On the unmodified PDMS surface, *P. aeruginosa* formed a thin and uniformly distributed biofilm. In contrast, on SA-coated PDMS surfaces, the biofilm appeared as dense, highly interconnected bacterial clusters (Fig. 3D). No differences in biofilm morphology were observed for *S. aureus* and *S. epidermidis* between SA pre-adsorbed and control surfaces, indicating that the effect of the pre-adsorbed SA layer on biofilm formation is also strain-dependent (Fig. S7).

Crystal violet (CV) staining was also employed to evaluate the biofilm formation capacity of the three bacterial strains on PDMS surface. Unlike CLSM analysis, CV stains all biofilm components, including bacterial cells and extracellular polymeric substances (EPS), enabling quantification of total biofilm biomass. However, CV quantification indicated that the presence of the SA layer did not alter the total biofilm biomass of any of the three strains after 24 h of incubation (Fig. S8). This suggests that the increased biofilm formation observed by CLSM for *P. aeruginosa* on SA-coated surfaces was due to a higher density of adhered bacterial cells rather than an increase in EPS production. Alternatively, the CV assay may lack the sensitivity to detect subtle differences in biofilm biomass, particularly in the case of *P. aeruginosa*, a species known for its limited ability to form robust biofilms at the solid-liquid interface [33].

As highlighted in various studies, biofilm bacterial density and structure are influenced by several factors, such as nutrient availability, *quorum sensing*, cell membrane appendages, and specific virulence genes in *P. aeruginosa* [[34], [35], [36]]. Notably, increasing evidence indicates that *P. aeruginosa* can sense and respond to surfaces through dedicated regulatory pathways that adjust its physiological state [[37], [38], [39]]. These findings suggest that, beyond altering surface physicochemical properties, SA pre-adsorption may exert a more complex effect on the physiology of surface-adherent bacteria. This response may play a significant role in the survival of the bacteria within the host environment and in its ability to resist the host's immune defences [40]. The following sections examine whether the altered biofilm morphology of *P. aeruginosa* on an SA-pre-adsorbed surface impacts macrophage-mediated bacterial killing. Additionally, we employed a proteomic approach to investigate how SA pre-adsorption influences the physiological responses of *P. aeruginosa*.

### 3.4 P. aeruginosa biofilms on the serum albumin-adsorbed surface show increased resistance to macrophage-mediated killing

**Fig. 4:**
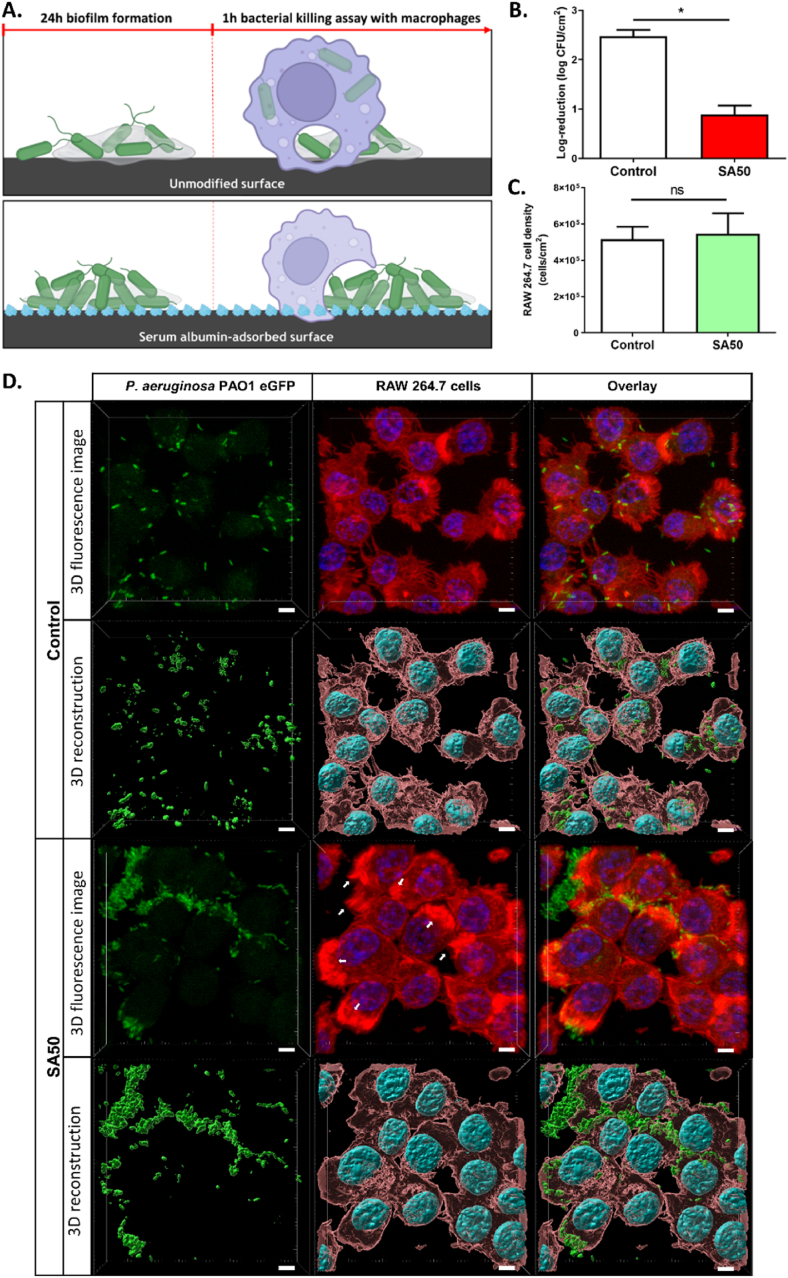
**Macrophage-mediated killing assay of 24 h *P. aeruginosa* biofilms pre-formed on PDMS surfaces.** (**A**) Schematic illustration of the bacterial killing experiment using murine RAW 264.7 macrophages (**B**) Percentage of *P. aeruginosa* bacteria remaining on unmodified PDMS (Control) and serum albumin-adsorbed surfaces (SA50) after 1 h of macrophage-mediated killing. (**C**) Density of RAW 264.7 cells on unmodified PDMS (Control) and serum albumin-adsorbed surfaces (SA50). (**D**) Representative confocal fluorescence images and 3D reconstruction images of *P. aeruginosa* biofilms on PDMS surfaces (Control and SA50) after 1 h of exposure to murine RAW 264.7 macrophages. *P. aeruginosa* cells were labeled with eGFP (green), actin was stained with Rhodamine-phalloidin (red), and nuclei were counterstained with DAPI (blue). White arrows highlight regions of pronounced actin accumulation at the site of bacterial encounter. Data are presented as mean ± SEM; n = 4; ns: not significant; ∗p < 0.05. Scale bar: 5 μm. (For interpretation of the references to colour in this figure legend, the reader is referred to the Web version of this article.)

Planktonic bacteria tend to adhere to surfaces and form biofilms, which in turn provide them with protection against both antimicrobial agents and the host immune system [41]. Given the structural differences observed in P. aeruginosa biofilms formed on serum albumin-adsorbed surfaces, we investigated whether these changes affect their susceptibility to macrophage-mediated killing. To examine this, 24 h P. aeruginosa biofilms pre-formed on serum albumin-pre-coated (SA50) and unmodified PDMS surfaces (Control) (Fig. 4A) were challenged to murine RAW 264.7 macrophages. Before co-culture, to assess any potential impact of the SA layer on macrophage activity, both surface types were tested for biocompatibility with eukaryotic cells, including murine RAW 264.7 macrophages and human lung epithelial A549 cells. After 24 h of incubation, no significant differences were observed in cell viability, metabolic activity, or morphology, indicating that the SA layer did not affect eukaryotic cells under the tested conditions (Fig. S9).

The macrophage concentration was standardized at 7 × 10^5^ cells per surface, corresponding to 100 % confluence. After a 1 h co-culture, adherent bacteria were detached by sonication and quantified by plating on agar. As shown in Fig. 4B, macrophage treatment of the control surface resulted in a 99.64 % reduction in bacterial load (2.5-log reduction) compared to the initial level. In contrast, biofilms on SA50 surfaces showed only an 86.32 % reduction (0.9-log reduction) after macrophage exposure. The higher level of bacterial clearance on the control surface suggests that most bacteria were either successfully engulfed and killed by macrophages or removed during the macrophage colonization process.

To further investigate macrophage interactions with the biofilms, fluorescence microscopy was used to assess both macrophage adhesion and phagocytic activity on the two surfaces. Quantification of adherent macrophages revealed no significant difference in cell density between the surfaces (Fig. 4C), indicating that both substrates supported efficient macrophage attachment, an essential prerequisite for effective phagocytosis. On the control surface, most *P. aeruginosa* cells appeared to be successfully internalized and were predominantly located within macrophages (Fig. 4D). In contrast, on the SA50 surface, macrophages showed limited ability to fully internalize the bacteria, particularly the microcolony structures. A pronounced accumulation of actin at macrophage-bacteria contact sites (indicated by white arrows) was observed, suggesting the formation of phagocytic cups around the bacterial targets. Similar findings have been reported in studies investigating the influence of bacterial morphology on phagocytosis [42,43]. For example, successful engulfment of filamentous *E. coli* (>10 μm) requires extensive cytoskeletal remodeling to form a complete phagocytic cup around the elongated bacteria, leading to slower internalization and reduced phagocytic efficiency [42]. This mechanism may help explain the significantly lower phagocytosis observed for biofilms pre-formed on serum albumin-coated surfaces compared to control surfaces.

These findings suggest that the adsorption layer of serum albumin not only influences *P. aeruginosa* adhesion and biofilm formation but also indirectly impacts the effectiveness of host immune defenses in clearing bacteria from the surface. Studies on the interactions between bacteria, host immune cells, and biomaterial surfaces have been widely reported in the literature [7,[44], [45], [46]]. However, most of these investigations have focused on illustrating the “race for the surface” between bacterial pathogens and host cells, which ultimately shapes the clinical outcome of biomaterial-associated infections. This study adds a novel perspective by showing that biofilm morphology, shaped by the properties of the underlying material, may also modulate the bactericidal efficiency of adherent macrophages.

### 3.5 Pre-adsorbed serum albumin layer alters the physiological state of adhered P. aeruginosa on the surface

**Fig. 5:**
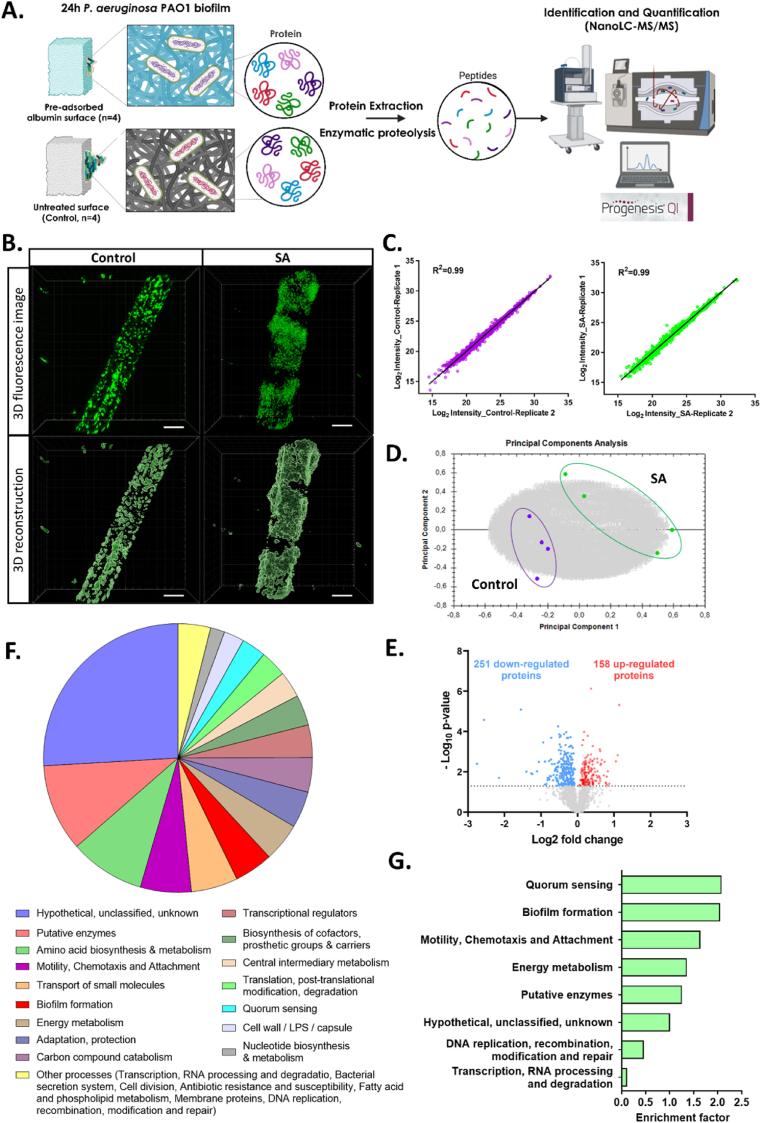
**Proteome analysis of adhered *P. aeruginosa* on pre-adsorbed-serum albumin surface** (**A**) Schematic of the experimental process showing the collection of protein samples from adhered bacteria for proteomic analysis. (**B**) Representative confocal images (top) and 3D reconstructions (bottom) of *P. aeruginosa* biofilms on unmodified glass wool fibers (Control) and SA-pre-adsorbed glass wool fibers (SA) after 24 h of incubation. Scale bar: 10 μm. (**C**) Correlation intensity in proteomic experiments between biological replicates (Replicates 1 and 2) for the control group (left) and the SA group (right). (**D**) Principal component analysis (PCA) showing the distribution of biological replicates of SA (n = 4) and control condition (n = 4) from their molecular profiles. (**E**) Volcano plot with cut-off *p*-value = 0.05 showing the differential expression of proteins in 24 h *P. aeruginosa* biofilms formed on SA-pre-adsorbed surfaces compared to control surfaces. Up-regulated and down-regulated proteins are highlighted in red and blue, respectively. (**F**) Classification of differentially expressed proteins extracted from adhered *P. aeruginosa* on SA and the control group according to their biological processes using PseudoCAP function categories. (**G**) Bar plot showing the biological enrichment of the differentially expressed proteins between the SA group and the control group. Enrichment analysis based on PseudoCAP function categories was conducted using Fisher's exact test in “Perseus” software, with a *p*-value threshold set at 0.05. The enrichment score of each pathway term is defined as the ratio of the DEP ratio (differentially expressed protein number annotated in this pathway to all proteins annotated in this pathway) and the background ratio (differentially expressed protein number to identified protein number). (For interpretation of the references to colour in this figure legend, the reader is referred to the Web version of this article.)

To better understand the impact of the pre-adsorbed SA layer on *P. aeruginosa*, we performed a quantitative proteomic study by a label-free approach on adhered cells (Fig. 5A). In this experiment, glass wool fibers were used as the substrate for biofilm formation instead of PDMS to ensure sufficient bacterial biomass for proteomic analysis [47]. Considering the significant influence of SA50 on the density and structure of *P. aeruginosa* biofilms, this condition was selected for proteomic analysis. After 24 h of incubating *P. aeruginosa* on control surfaces (n = 4) and SA-pre-adsorbed surfaces (n = 4), bacterial adhesion on the glass wool fibers was confirmed by CLSM (Fig. 5B). Cell counting and biovolume quantification of *P. aeruginosa* biofilm on glass wool fibers revealed a significant increase in adhered cells in the SA group, consistent with the previously observed phenotype on PDMS surfaces (Fig. S10). These adhered cells were then harvested, lysed, and their peptides were analyzed by high-resolution LC-MS/MS spectrometry, as described in Fig. 5A. The scatter plot of peptide MS intensity results demonstrated good reproducibility among the biological replicates, with a correlation coefficient > 0.99 (Fig. 5C). Interestingly, principal component analysis (PCA) of the identified proteins revealed a clear proteomic distinction between the SA-pre-adsorbed and control samples, emphasizing a significant physiological difference in adhered *P. aeruginosa* under these two conditions (Fig. 5D).

Proteome analysis identified 1986 proteins in *P. aeruginosa* cells for label-free quantification. Among these, 251 proteins were down-regulated and 158 were up-regulated when the bacteria were exposed to the pre-adsorbed SA layer, based on an adjusted *p*-value of less than 0.05 (Fig. 5E). Fig. 5F categorizes these differentially represented proteins from the SA group and the control group according to their biological processes, using the PseudoCAP functional classification [48]. Beyond the proteins of unknown function and putative enzymes, many are involved in amino acid biosynthesis and metabolism, motility, chemotaxis, attachment, small molecule transport, and biofilm formation. The complete list of identified and differentially expressed proteins can be found in the Supplementary Table S1.

To enable further biological interpretation of the data, differentially expressed protein pathways were classified using enrichment analysis employing a Fisher's exact test with a *p*-value threshold of 0.05 (Supplementary Table S2). This analysis examines whether the differentially expressed proteins in each biological process differ from a uniform distribution. Fig. 5G shows significantly enriched pseudoCAP terms for differentially expressed proteins in the SA group. Higher abundance was observed for proteins involved in *quorum sensing* (QS), biofilm formation, motility, chemotaxis, and attachment. These results align with our initial hypothesis, as the density and structure of the biofilm are primarily regulated by these biological pathways.

**Fig. 6:**
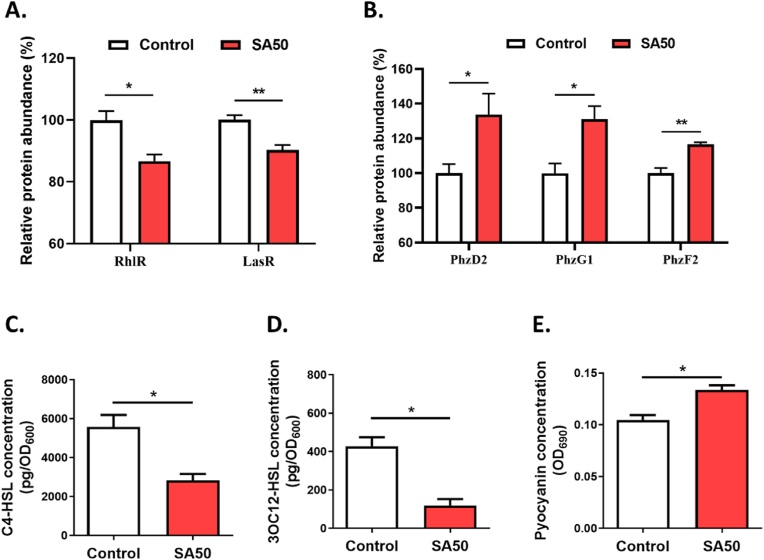
**Pre-adsorption of serum albumin onto the surface affects the *quorum sensing* system of adhered *P. aeruginosa***. Relative abundance of *quorum sensing* regulators RhlR and LasR (**A**) and phenazine biosynthetic proteins PhzD2, PhzG1, and PhzF2 (**B)** in *P. aeruginosa* 24 h biofilms formed on SA-pre-adsorbed and control surfaces. Quantification of C4-HSL (**C**), 3-oxo-C12HSL (**D**), and pyocyanin (**E**) in the surrounding media after 24 h of incubating, with or without SA pre-adsorption. Data are presented as mean ± SEM; n = 4; ∗p < 0.05.

QS is a mechanism of inter-bacterial communication that relies on the production and detection of signaling molecules called autoinducers [35]. When the concentration of autoinducers reaches a threshold, they bind to their respective receptors, modulating the activity of various genes responsible for virulence-related phenotypes. In *P. aeruginosa*, the QS network consists of three interrelated systems: the Las system, the Rhl system, and the Pqs system. Interestingly, both RhlR and LasR proteins were found to be down-expressed in the SA group (Fig. 6A). This suggests that the QS system could be disrupted in the presence of the SA-pre-adsorbed layer. It is well known that the Las and Rhl systems each feature an AHL synthase (LasI and RhlI) responsible for producing autoinducers, N-(3-oxododecanoyl)-L-homoserine lactone (3-oxo-C12-HSL) and N-butanoyl-L-homoserine lactone (C4-HSL), respectively. These autoinducers then interact with their cognate transcriptional regulators, LasR and RhlR, leading to the activation or repression of multiple genes regulated by QS. To verify the hypothesis that the SA layer could affect the QS systems, these two autoinducers were extracted and quantified from the media surrounding the SA-pre-adsorbed and control surfaces using LC-MS/MS. As expected, while the density of planktonic *P. aeruginosa* around the surfaces remained unchanged by the albumin layer, the levels of the QS autoinducers C12-HSL and C4-HSL were significantly reduced in the surrounding medium of the SA-pre-adsorbed surface (Fig. 6CD).

Alongside the dysregulation of LasR and RhlR, we also observed an upregulation of PhzD2, PhzG1, and PhzF2 in the SA group (Fig. 6B). The phz operons are regulated by the QS systems and encode the enzymes required for synthesizing phenazine compounds, key metabolic products in *P. aeruginosa*. Among these, pyocyanin is a well-studied phenazine compound implicated in the virulence of *P. aeruginosa* infections [49]. Consistent with the proteomic analysis, the pyocyanin production increased in the groups with pre-adsorbed SA surfaces compared to the control group (Fig. 6E).

It should be noted that inhibiting QS is considered a promising strategy for targeting the virulence factors of *P. aeruginosa*, as well as pathogenic bacteria. So much research aims to develop anti-QS surfaces as an alternative to traditional antibacterial surfaces, yielding impressive results [3,50]. In this context, the anti-QS capability of the adsorbed-SA layer could represent a natural defence mechanism by which serum limits the virulence of *P. aeruginosa*. Similarly, pre-treating medical implants with SA before introducing them into the body may offer similar benefits in reducing bacterial virulence and infection risk.

However, it is important to emphasize that QS-regulated virulence factors are not the only determinants of *P. aeruginosa* infection risk. The virulence of *P. aeruginosa* involves the coordinated expression of numerous determinant factors, each regulated by multiple interconnected systems. In our experiments, the dysregulation of the Phz proteins and pyocyanin production in response to SA-pre-adsorbed surfaces might occur independently of the Las and Rhl systems. Indeed, several studies have shown that even when QS transcriptional regulators like LasR or RhlR are inactivated, the expression of virulence factors in *P. aeruginosa* remains unaffected [51,52]. Additionally, a high prevalence of QS-silent *P. aeruginosa* strains is particularly common among cystic fibrosis patients [53].

Although we have not yet identified the exact mechanism by which the SA layer reduces QS-autoinducer concentrations in the surrounding environment, our observations are consistent with previous reports suggesting that albumin might act as a "sink" for HSLs [54,55]. Smith et al. demonstrated that high albumin concentrations in culture media can reduce the virulence and competitive ability of *P. aeruginosa* in a polymicrobial interaction model [40]. Since polymicrobial interactions can influence the survival of different species, variations in albumin concentrations at infected sites could have significant clinical implications for polymicrobial infections. In our study, the effect of the adsorbed albumin layer on multi-bacterial interactions was not examined. However, we propose that variations in SA concentration around implanted medical devices, coupled with differing levels of albumin adsorption on various material surfaces, could similarly affect the natural selection of pathogenic species on these surfaces [56].

In addition to quorum sensing, we observed the dysregulation of various proteins associated with bacterial motility, surface adhesion (Table 1) and biofilm formation (Table 2). Notably, flagellar proteins, including FliD, FleN, and FliM, along with several proteins involved in flagella-mediated chemotaxis pathways (PA0179, PA1930, PA2920, PA2788, McpB, CheR1, PA2867, CheA, MotB, and PA1057), was downregulated in the SA group. Flagella are rotating appendages that P. aeruginosa uses to propel its cell body forward (swimming motility). Beyond the role in movement, increasing evidence suggests that flagella play a key role in the initial adhesion of bacteria to surfaces [57]. Indeed, active flagella help bacteria navigate through liquid environments to reach the surface, overcoming the electrostatic repulsion barrier created by surface charges [58,59]. Mutants lacking flagella are poor colonizers of solid surfaces, as they can only rely on free diffusion to reach the surface [58]. The direction of flagellar motor rotation is controlled by the chemotaxis signalling system, which includes four modules: sensor, transduction, actuator, and integral feedback [60]. Interestingly, most proteins involved in these pathways exhibited reduced expression. This suggests that cells adhered to the SA-adsorbed surface after 24 h of incubation may have reached a stage of irreversible attachment, during which stable connections between the surface and the cell body are established [58].

**Table 1:** Differentially regulated proteins related to motility and attachment processes in *P. aeruginosa* between adhered cells on SA-pre-adsorbed and control surfaces.

	Accession	*Gene name*	Product name	Expression in the SA-pre-adsorbed surface	Fold change	*P*-value
**Motility, Chemotaxis and Attachment**	PA0179		probable two-component response regulator	Down-regulated	1.70	2.37E-02
	PA1930		probable chemotaxis transducer	Down-regulated	1.63	4.11E-03
	PA2920		probable chemotaxis transducer	Down-regulated	1.58	1.44E-02
	PA2788		probable chemotaxis transducer	Down-regulated	1.53	7.19E-03
	PA0176	***mcpB***	methyl-accepting chemotaxis protein	Down-regulated	1.50	5.97E-03
	PA3348	***cheR1***	probable chemotaxis protein methyltransferase	Down-regulated	1.40	4.41E-04
	PA1094	***fliD***	flagellar capping protein FliD	Down-regulated	1.35	4.74E-03
	PA2867		probable chemotaxis transducer	Down-regulated	1.34	4.50E-02
	PA1458	***cheA***	probable chemotaxis signal transduction histidine kinase	Down-regulated	1.26	4.74E-02
	PA4953	***motB***	chemotaxis protein MotB	Down-regulated	1.21	1.92E-02
	PA0355	***pfpI***	protease PfpI	Down-regulated	1.20	1.14E-03
	PA1454	***fleN***	flagellar synthesis regulator FleN	Down-regulated	1.14	1.77E-03
	PA3702	***wspR***	WspR	Down-regulated	1.12	3.80E-02
	PA1801	***clpP***	ClpP	Down-regulated	1.10	9.17E-03
	PA1443	***fliM***	flagellar motor switch protein FliM	Down-regulated	1.10	6.48E-04
	PA5040	***pilQ***	Type 4 fimbrial biogenesis outer membrane protein PilQ precursor	Up-regulated	1.35	1.53E-02
	PA4969	***cpdA***	Cyclic AMP (cAMP) Phosphodiesterase. CpdA	Up-regulated	1.34	5.23E-04
	PA4550	***fimU***	type 4 fimbrial biogenesis protein FimU	Up-regulated	1.33	2.80E-03
	PA0415	***chpC***	probable chemotaxis protein	Up-regulated	1.31	2.02E-02
	PA0414	***chpB***	probable methylesterase	Up-regulated	1.30	6.53E-03
	PA1822	***fimL***	hypothetical protein	Up-regulated	1.25	3.75E-03
	PA4554	***pilY1***	type 4 fimbrial biogenesis protein PilY1	Up-regulated	1.22	5.54E-03
	PA3805	***pilF***	type 4 fimbrial biogenesis protein PilF	Up-regulated	1.17	1.11E-02
	PA0395	***pilT***	twitching motility protein PilT	Up-regulated	1.12	4.02E-02
	PA5112	***estA***	esterase EstA	Up-regulated	1.11	3.54E-02

**Table 2:** Differentially regulated proteins related to biofilm formation processes in *P. aeruginosa* between adhered cells on SA-pre-adsorbed and control surfaces.

	Accession	Gene name	Product name	Expression in the SA-pre-adsorbed surface	Fold change	*P*-value
**Biofilm formation**	PA1863	***modA***	molybdate-binding periplasmic protein precursor ModA	Down-regulated	5.90	2.63E-05
	PA0852	***cbpD***	chitin-binding protein CbpD precursor	Down-regulated	2.92	8.11E-06
	PA2371	***clpV3***	ClpV3	Down-regulated	2.64	9.76E-03
	PA2939	***paaP***	probable aminopeptidase. quorum-sensing-regulated enzyme	Down-regulated	2.36	1.28E-02
	PA0090	***clpV1***	ClpV1	Down-regulated	1.46	3.60E-03
	PA2237	***pslG***	PslG	Down-regulated	1.44	2.86E-02
	PA3346	***hsbR***	two-component response regulator	Down-regulated	1.34	3.84E-02
	PA0423	***pasP***	PasP	Down-regulated	1.32	2.34E-02
	PA4446	***algW***	AlgW protein	Down-regulated	1.32	2.00E-02
	PA0085	***hcp1***	Hcp1	Down-regulated	1.31	2.15E-02
	PA0083	***tssB1***	TssB1	Down-regulated	1.29	4.15E-02
	PA2235	***pslE***	PslE	Down-regulated	1.28	1.31E-02
	PA2302	***ambE***	AmbE	Down-regulated	1.24	2.43E-02
	PA4457	*kdsD*	arabinose-5-phosphate isomerase KdsD	Down-regulated	1.13	2.48E-03
	PA5261	***algR***	alginate biosynthesis regulatory protein AlgR	Down-regulated	1.10	2.98E-02
	PA2586	***gacA***	response regulator GacA	Down-regulated	1.22	2.11E-04
	PA2234	***pslD***	PslD	Down-regulated	1.18	7.01E-03
	PA2232	***pslB***	PslB	Down-regulated	1.11	3.02E-02
	PA0649	***trpG***	anthranilate synthase component II	Down-regulated	1.10	2.45E-03

Additionally, the expression of two protease-related proteins, PfpI and ClpP, involved in the swarming motility of *P. aeruginosa*, was also reduced in this condition. Among them, PfpI is a QS-dependently secreted protein factor that plays a significant role in the infection process by disrupting the host immune system during infection [61,62].

Meanwhile, the type IV fimbrial (pili) proteins (PilQ, CpdA, FimU, ChpC, ChpB, FimL, PilY1, PilF and PilT) were overexpressed in bacteria adhering to a surface pre-adsorbed with SA. Type IV pili are external appendages located at the poles of *P. aeruginosa*. They act as key virulence factors, critically involved in host tissue colonization, biofilm formation, and a specialized surface translocation mechanism known as twitching motility [63]. These filamentous structures help bacteria transition from a single-layer arrangement to forming microcolonies by overcoming repulsive forces upon contact with solid surfaces, ultimately shaping the biofilm morphology [64]. In addition, twitching motility has been shown to promote active biofilm expansion by *P. aeruginosa* along the surface of indwelling catheters [65,66]. Numerous studies have shown that mutants lacking Type IV pili can adhere to surfaces and form a monolayer but cannot aggregate into more complex structures [[67], [68], [69]]. This observation is consistent with our results, which indicate that bacterial cells have reached a late stage in the adhesion process, explaining the significant number of adhered cells found on the SA-pre-adsorbed surface (Fig. 3C). Additionally, these findings may account for the differences in the biofilm structure of *P. aeruginosa* on PDMS surfaces, where bacterial cells form denser aggregates on the SA-coated surface compared to the control surface (Fig. 3D).

Interestingly, the Chp system, particularly ChpC and ChpA, has been reported to play a role in sensing host-derived signals such as serum albumin, mucin, and oligopeptides [[70], [71], [72]]. These signals trigger an increase in twitching motility, along with elevated levels of 3′,5′-cyclic adenosine monophosphate (cAMP) and Type IV pili. According to Nolan et al., ChpC can relay these environmental cues, including serum albumin, into the Chp system to regulate twitching motility-driven biofilm expansion [70].

After attachment, bacterial biofilms spread through surface motility and grow *via* EPS secretion [60]. While the extension and retraction of Type IV pili ensure bacterial movement parallel to the surface, EPS secretion provides a scaffold essential for forming stable surface biofilms. In our study, although cells that adhered to the SA-pre-adsorbed surface progressed further into the irreversible adhesion stage compared to those on the control surface, the expression of proteins involved in biofilm formation was reduced under these conditions. Specifically, the expression of proteins responsible for producing Psl and alginate, two of the three key EPS polysaccharides (PslG, PslE, PslD, PslB, AlgW and AlgR), was simultaneously down-regulated in biofilms formed on SA-pre-adsorbed surfaces (Table 2). Alginate is a high-molecular-weight polysaccharide composed of repeating units of mannuronic acid and guluronic acid. There is considerable evidence that alginate is a key EPS component contributing to antibiotic resistance and virulence of *P. aeruginosa*. In addition, Psl is a repeating pentamer of mannose, rhamnose, and glucose [73]. Together with the polysaccharide Pel, Psl forms a major part of the structural scaffold of biofilms on solid surfaces. Numerous previous studies demonstrated that Psl is also crucial in initiating biofilm growth and the development of new microcolonies, due to its role in adhesion. The reduced expression of proteins involved in EPS production may be linked to the anti-QS effect of the adsorbed SA layer, or the biofilms formed on the SA-coated surfaces may have reached a more mature state compared to the control group [35]. Indeed, through the enhanced Type IV pili production, biofilms formed on the SA-pre-adsorbed surface may require less involvement of EPS polysaccharide synthesis proteins compared to those formed on unmodified surfaces [74].

In addition to this explanation, we also considered whether the observed biofilm morphology (Fig. 3D) could result from passive bacterial aggregation induced by the presence of SA on the surface, independent of classical biofilm formation mechanisms. Bacteria are known to aggregate via a process called “depletion aggregation,” driven by entropic forces generated by abundant macromolecules (such as polymers, eDNA, and proteins) commonly found at chronic infection sites [75,76]. This aggregation mechanism does not depend on biofilm formation functions but can make bacteria more resistant to antibiotic killing [76]. To investigate this possibility, we performed time-lapse imaging of *P. aeruginosa* biofilm formation during the initial stages of adhesion. As shown in Fig. S11, no notable bacterial clustering was observed under either condition within our experimental setup. This result supports the interpretation that the observed biofilm morphology changes are more likely attributable to physiological changes in bacterial behavior rather than passive, driven physical interactions. Furthermore, we examined whether co-adsorption of SA with other serum proteins could influence the observed phenotype. Apo-transferrin, a serum protein known for its anti-adhesive and antibiofilm properties, was used as a control. However, the effect of SA on *P. aeruginosa* biofilm formation remained unchanged, indicating that the observed phenotype is specifically associated with SA and not influenced by co-adsorbed serum proteins (Fig. S12).

In addition to the repression of the EPS secretion process, several proteins closely associated with biofilm formation and the virulence of *P. aeruginosa* (such as ModA, PaaP, AmbE, and PasP) were found to be down-regulated in the SA group. Notably, proteins from the Type VI (ClpV3, ClpV1, Hcp1, TssB1) and Type II (CbpD) secretion systems, responsible for delivering effectors (toxins) to the extracellular milieu and host organisms, were also observed to be underexpressed. An interesting point is that the production of EPS and the aforementioned virulence factors has largely been shown to depend on QS systems [[77], [78], [79]]. This once again demonstrates the potential anti-QS ability of the SA-pre-adsorbed surface, contributing to the reduced biofilm formation and biofilm-associated virulence factors of *P. aeruginosa*.

**Fig. 7:**
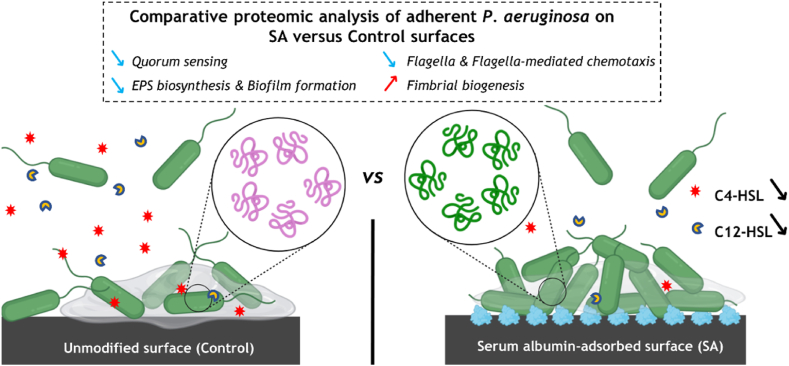
Schema illustrating the potential effect of serum albumin pre-adsorption onto the surface on *P. aeruginosa*.

In summary, our findings reveal a substantial impact of SA pre-adsorption on bacterial responses of *P. aeruginosa* during surface colonization, a phenomenon not previously reported. The most significant effects include changes in QS, biofilm formation, motility, and attachment (Fig. 7). The presence of a SA layer reduces the abundance of the Las and Rhl systems, along with their corresponding autoinducers, in the environment. A notable consequence of the QS inhibition is the reduced abundance of proteins involved in the production of the biofilm EPS matrix when bacteria adhere to the SA-pre-adsorbed surface. In addition to its impact on QS, bacterial adhesion to the SA-pre-adsorbed surface is promoted by the increased abundance of fimbrial proteins, which are crucial for the transition from motility to irreversible attachment. This observation may explain why *P. aeruginosa* tends to form a dense cellular aggregate on the SA surface, while only creating a monolayer on the PDMS control surface.

It should be noted that although the production of EPS is reduced when bacteria adhere to the SA surface, the high density of *P. aeruginosa* bacteria may hinder the bactericidal efficacy of antibiotics and the body's immune system. This is particularly important because, in recent years, SA coating has been proposed as a safe method to increase the biocompatibility of biomaterials and reduce the risk of infection [8]. While this approach may be effective for certain bacterial species or at specific stages of contamination (e.g., initial adhesion), it may lead to unintended consequences in other contexts, as demonstrated by the case of *P. aeruginosa* in this study. This also emphasizes that focusing solely on individual anti-virulence mechanisms, such as anti-adhesion or anti-biofilm strategies, or measuring isolated virulence factors in the environment may not lead to accurate conclusions about the efficacy of antimicrobial surfaces. Moreover, this study highlights the importance of proteomic approaches in exploring bacterial-surface interactions, offering important insights into the adaptive responses of bacteria to both host environments and foreign materials.

## 4 Conclusion

With ongoing advancements in biomaterials research, their application in tissue engineering and medical implants continues to expand. Beyond meeting the technical requirements of specific applications, optimizing material surfaces to resist infection during and after implantation is crucial for prolonging their longevity and preserving the patient's health. A key step in achieving this is understanding the molecular mechanisms by which bacteria interact with biomaterial surfaces. In this study, we investigated the impact of a SA-pre-adsorbed layer, the predominant plasma protein, on bacterial adhesion and biofilm formation of three nosocomial pathogens. First, we demonstrated that the effect of the SA-pre-adsorbed layer varies between bacterial species. While SA pre-adsorption altered the surface's physicochemical properties and reduced the initial adhesion of *S. aureus* and *S. epidermidis*, it did not significantly affect the subsequent biofilm formation. In contrast, SA pre-adsorption on the surfface increased bacterial density and altered the biofilm structure of P. aeruginosa. This alteration was associated with a marked decrease in the efficiency of macrophage-mediated bacterial clearance of *P. aeruginosa*.

In-depth biofilm proteomic analysis of *P. aeruginosa* biofilms formed on SA-pre-adsorbed surfaces revealed a disruption of the QS system and a reduction in the EPS production, two key factors in biofilm formation. However, the SA-pre-adsorbed surfaces increased the expression of fimbrial proteins, responsible for the transition from motility to irreversible attachment, generating an increase in the adherence ability. These proteomic findings, combined with phenotypic observations, suggest that biofilms formed on SA-coated surfaces may have reached a more mature state compared to those on unmodified surfaces.

Collectively, these results provide important insights into the cellular mechanisms of bacterial contamination on biomaterial surfaces, particularly regarding interactions with SA-pre-adsorbed surfaces. While further validation under physiologically relevant conditions (e.g., dual-species infections, dynamic flow environments, or *in vivo* models) is needed, our findings may help explain certain complex clinical observations related to device-associated infections. Notably, although serum albumin coatings have been proposed in recent years to enhance biomaterial biocompatibility, they may also increase the risk of biomaterial-associated infections in a species-dependent manner.

## CRediT authorship contribution statement

**Hung Le:** Writing – review & editing, Writing – original draft, Methodology, Investigation, Formal analysis, Conceptualization. **Marie Droniou:** Writing – review & editing, Writing – original draft, Methodology, Investigation, Formal analysis. **Lisa Wallart:** Writing – review & editing, Writing – original draft, Methodology, Investigation, Formal analysis. **Laurent Coquet:** Writing – review & editing, Writing – original draft, Methodology, Investigation, Formal analysis. **Pascal Thebault:** Writing – review & editing, Writing – original draft, Methodology, Investigation, Formal analysis. **Clément Guillou:** Writing – review & editing, Writing – original draft, Methodology, Investigation, Formal analysis, Conceptualization. **Pascal Cosette:** Writing – review & editing, Writing – original draft, Validation, Supervision, Methodology, Conceptualization.

## Declaration of competing interest

The authors declare the following financial interests/personal relationships which may be considered as potential competing interests: H Le reports financial support was provided by "Vaindre la Mucoviscidose" and "Grégory Lemarchal" Associations. M Droniou reports financial support was provided by University of Rouen Normandy. P Cosette reports financial support was provided by Normandy Region. P Cosette reports financial support was provided by Europe. If there are other authors, they declare that they have no known competing financial interests or personal relationships that could have appeared to influence the work reported in this paper.

## Data Availability

Data will be made available on request.
