# Supplementary material for: Pre-adsorption of serum albumin on biomaterial surfaces modulates bacteria-surface interactions and alters bacterial physiological responses
Source: Mater Today Bio. 2025 Aug 30;35:102254. doi: 10.1016/j.mtbio.2025.102254 (PMC12859551; doi:10.1016/j.mtbio.2025.102254)
Supplement: Multimedia component 1 [file mmc1.docx]

**Supplementary material**

**Pre-adsorption of serum albumin on biomaterial surfaces modulates bacteria-surface interactions and alters bacterial physiological responses**

*Hung Le^1,2^*^*^*_,_ Marie Droniou^1^, Lisa Wallart^1^, Laurent Coquet^1,2^, Pascal Thebault^1^, Clément Guillou^,2^*^*^ and *Pascal Cosette^1^*^,^*^2^*^*^

^1^ Univ Rouen Normandie, INSA Rouen Normandie, CNRS, Normandie Univ, PBS UMR 6270, Rouen, France

^2^ Univ Rouen Normandy, INSERM US 51, CNRS UAR 2026, HeRacLeS PISSARO, Rouen, France

^*^ *Correspondence authors*


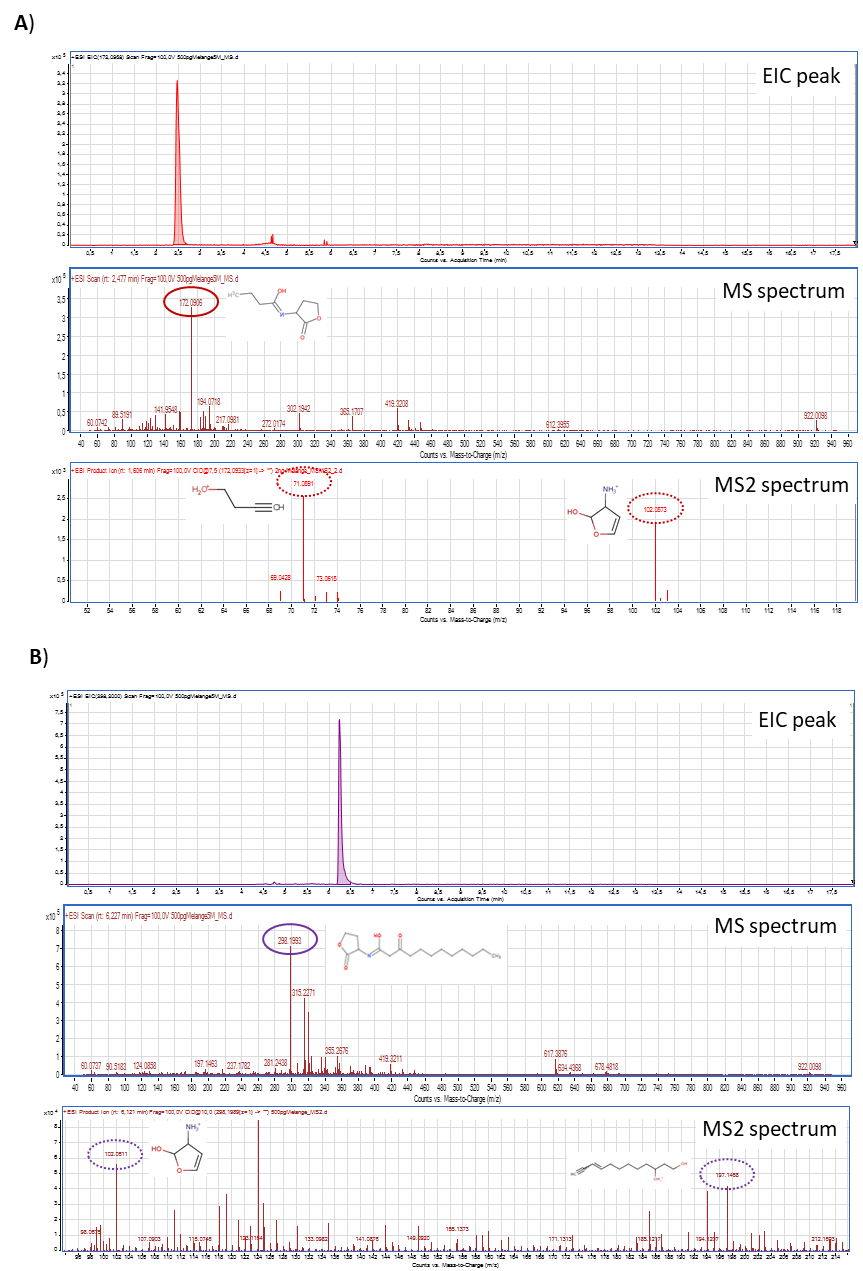


**Figure S1**: EIC peaks, MS and MS/MS spectra of C4-HSL (**A**) and 3-oxo-C12-HSL (**B**) : the full circles in the MS spectra show the expected m/z ions for the 2 AHLs and the dotted circles in the MS2 spectra show the characteristic fragment ions.


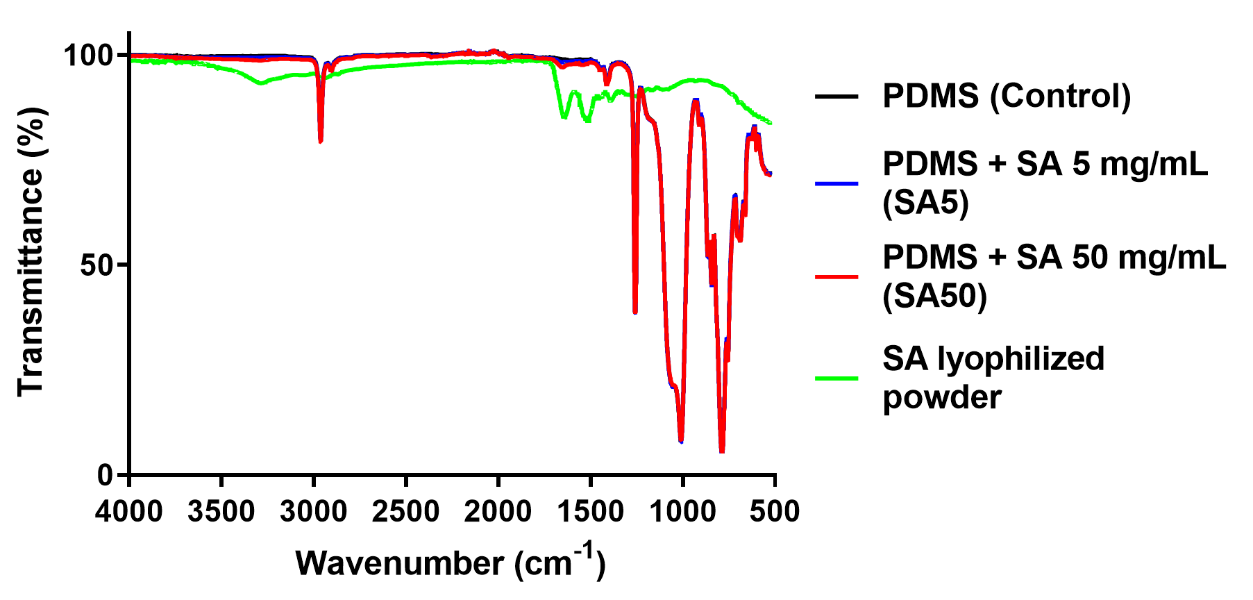


**Figure S2**. Full FTIR spectra of the unmodified PDMS surface (Control), PDMS surfaces pre-adsorbed with SA at concentrations of 5 mg/mL (SA5) and 50 mg/mL (SA50), and pure SA powder.


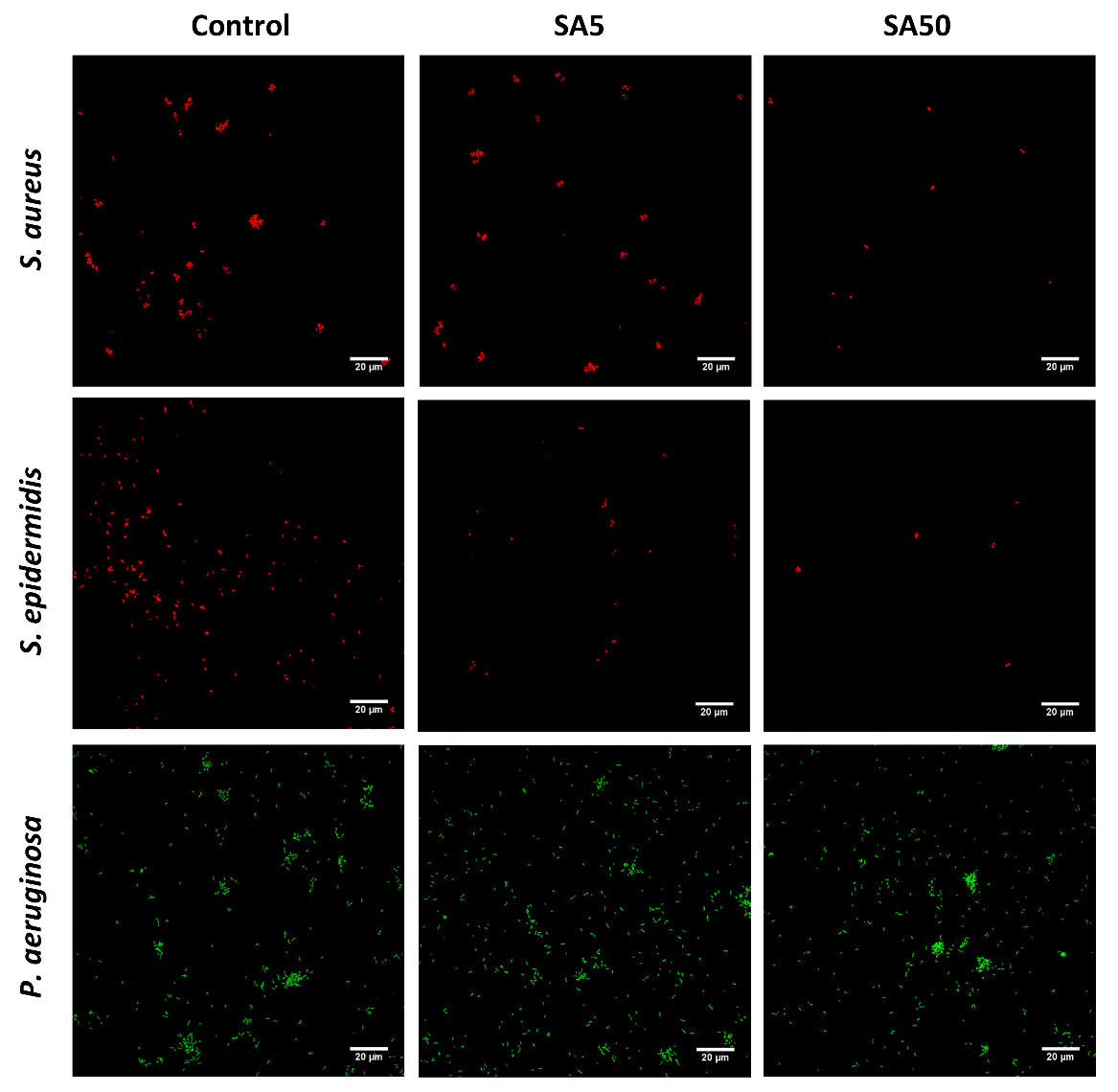


**Figure. S3**. Representative wild-field fluorescence images showing bacterial adhesion on unmodified PDMS surfaces (Control) and SA-pre-adsorbed surfaces (SA5 and SA50) after 2 h of incubation; Scale bar: 20 μm.


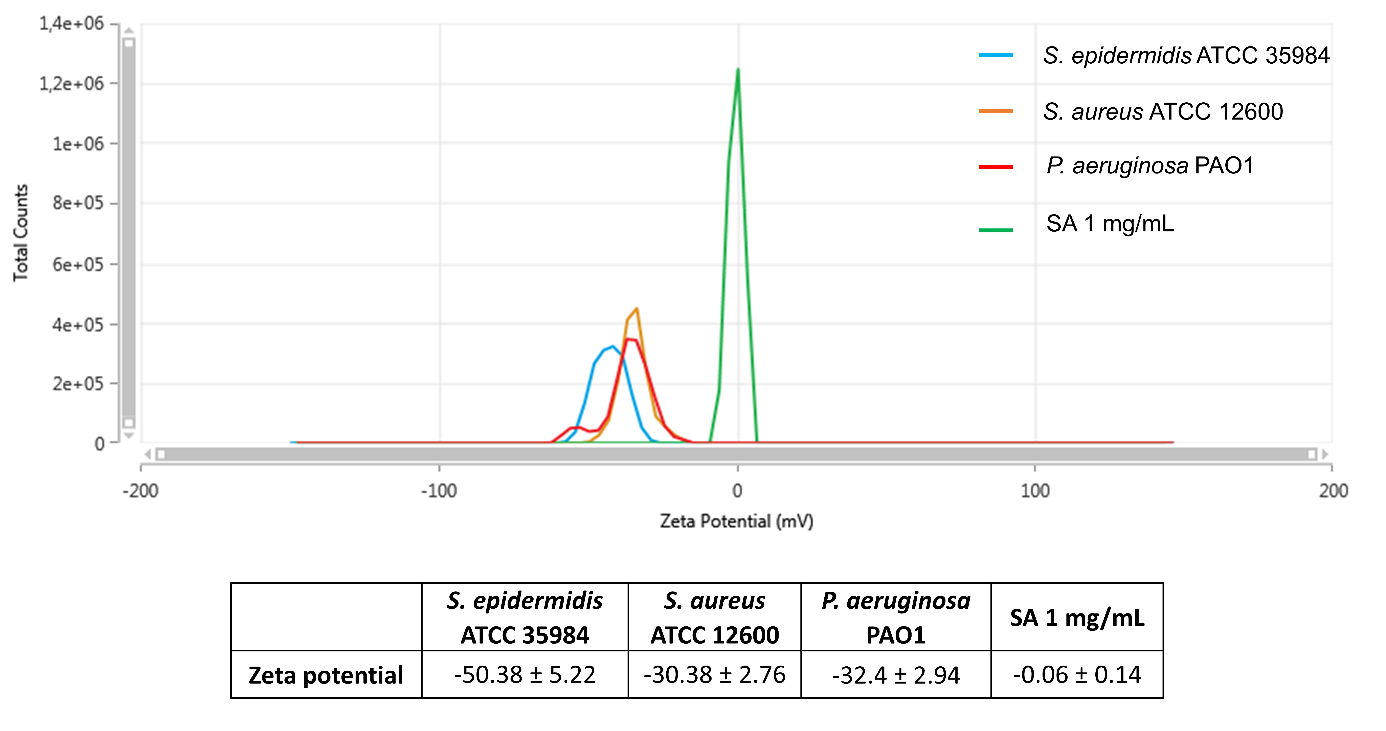


**Figure S4**. Surface charge (zeta potential) of *S. epidermidis* ATCC 35984, *S. aureus* ATCC 12600, *P. aeruginosa* PAO1, and serum albumin (SA) in water. Zeta potential was measured using electrophoretic light scattering (ELS) with a Zetasizer Ultra (Malvern Panalytical, Worcester, UK). To ensure optimal measurement conditions, overnight bacterial precultures were diluted in deionized water to a concentration of approximately 107 CFU/mL. The SA50 solution was similarly diluted in deionized water to a final concentration of 1 mg/mL.. All measurements were conducted at 25 °C following a 30-second temperature stabilization. Data are presented as mean ± SD; n = 3.


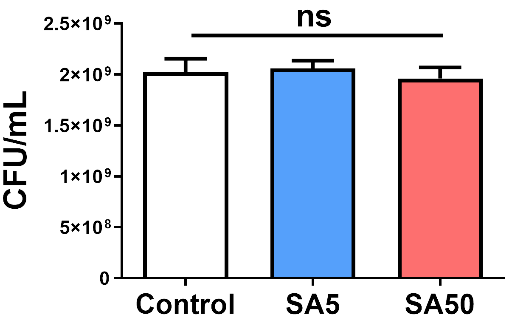


**Figure S5**. Quantification of planktonic *P. aeruginosa* cell around unmodified PDMS surfaces (Control) and SA-pre-adsorbed surfaces (SA5 and SA50) after 24 h of incubation. Data are represented as mean ± SEM; n=4, ns: not significant.


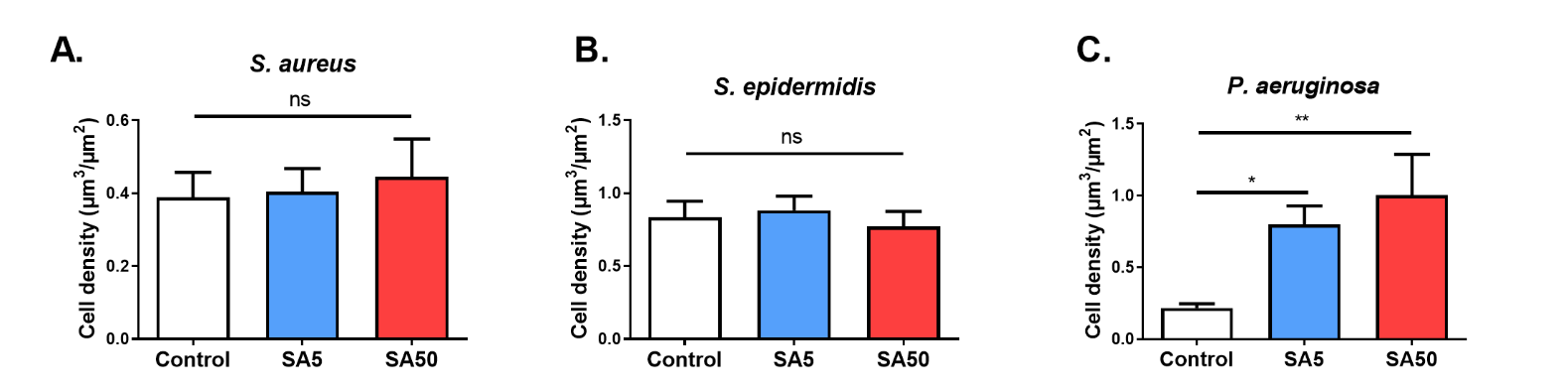


**Figure S6**. Quantification of biofilm biovolume by confocal microscopy for *S. aureus* (A), *S. epidermidis* (B), and *P. aeruginosa* (C) biofilms on control, SA5-, and SA50 surfaces. Gram-positive bacteria (*S. aureus* and *S. epidermidis*) were stained with hexidium iodide (excitation/emission: 488/600 nm), while the Gram-negative *P. aeruginosa* was stained with SYTO 9 (488/520 nm); Results are expressed as µm^3^/µm^2^. Data are represented as mean ± SEM; n = 4, ns: not significant; *p < 0.05; **p < 0.01


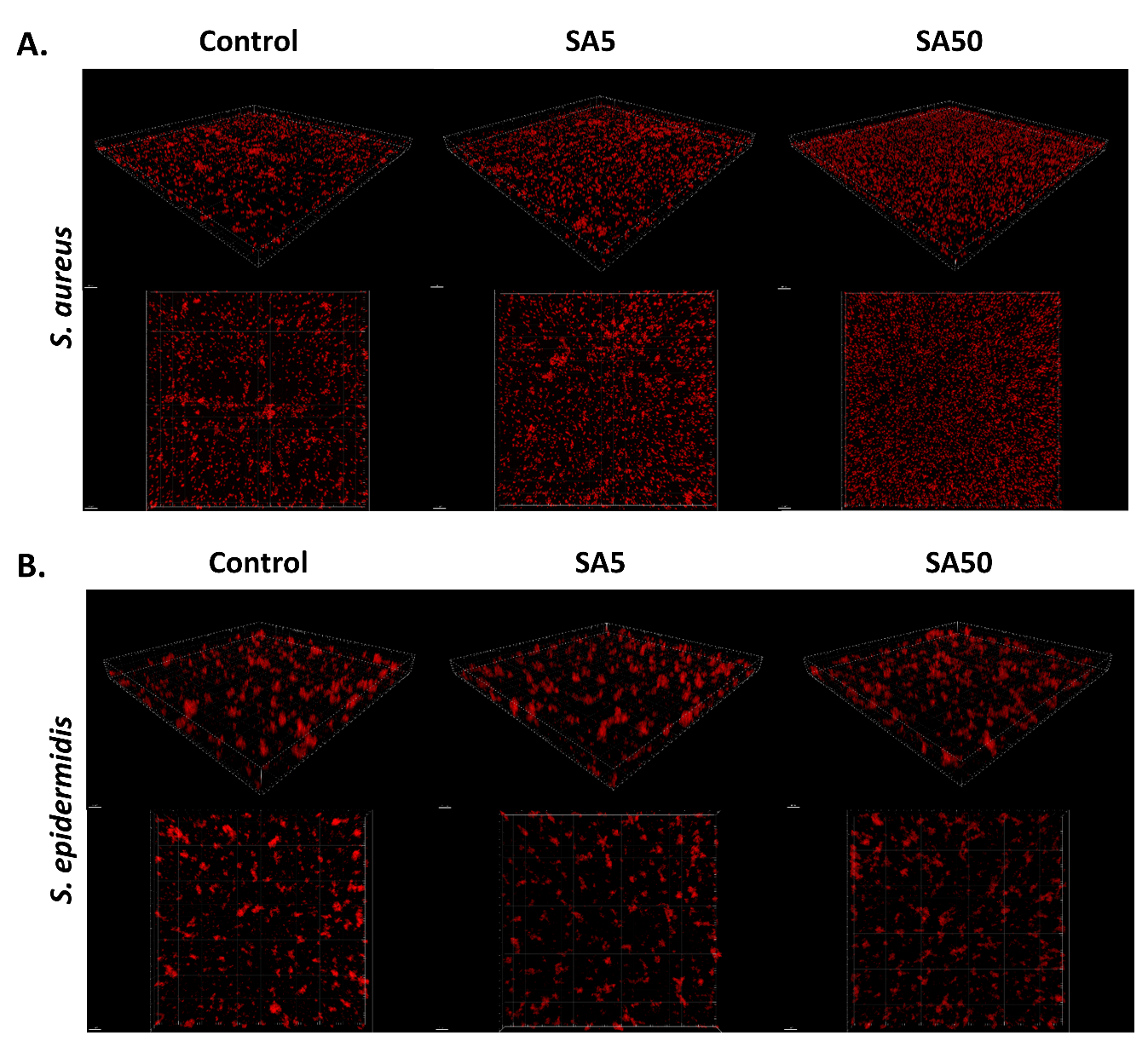


**Figure S7**. Representative confocal microscopy images of biofilm formation by *S. aureus* (**A**) and *S. epidermidis* (**B**) on unmodified PDMS surfaces (Control) and SA-pre-adsorbed surfaces (SA5 and SA50). Adherent cells were stained with hexidium iodide (excitation/emission: 488/600 nm); Scale bar: 10 μm.


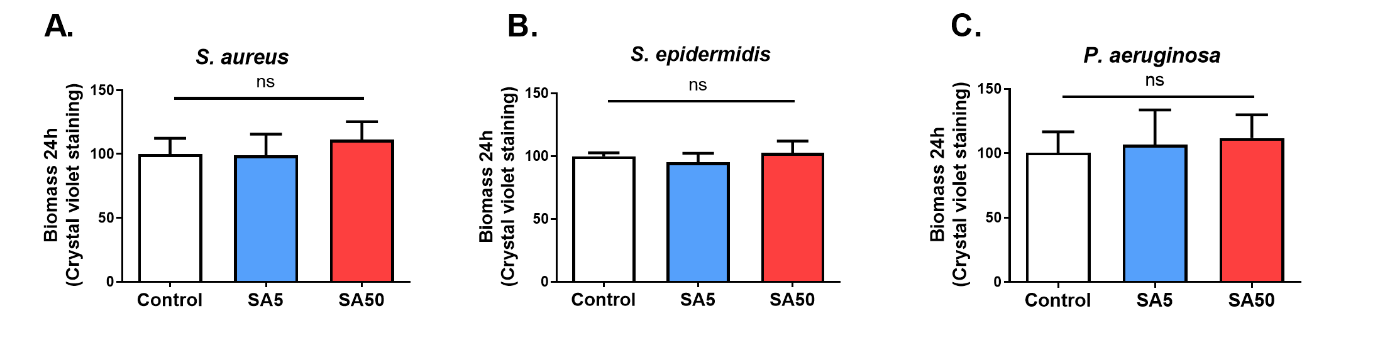


**Figure S8**. Relative biomasses measured from *S. aureus* (**A**), *S. epidermidis* (**B**) and *P. aeruginosa* (**C**) biofilms on PDMS surfaces after crystal violet staining. The results are expressed as a percentage of biofilm biomass (OD580), with the control group set to 100%. Data are represented as mean ± SEM; n=4, ns: not significant; **p* < 0.05; ***p*<0.01.


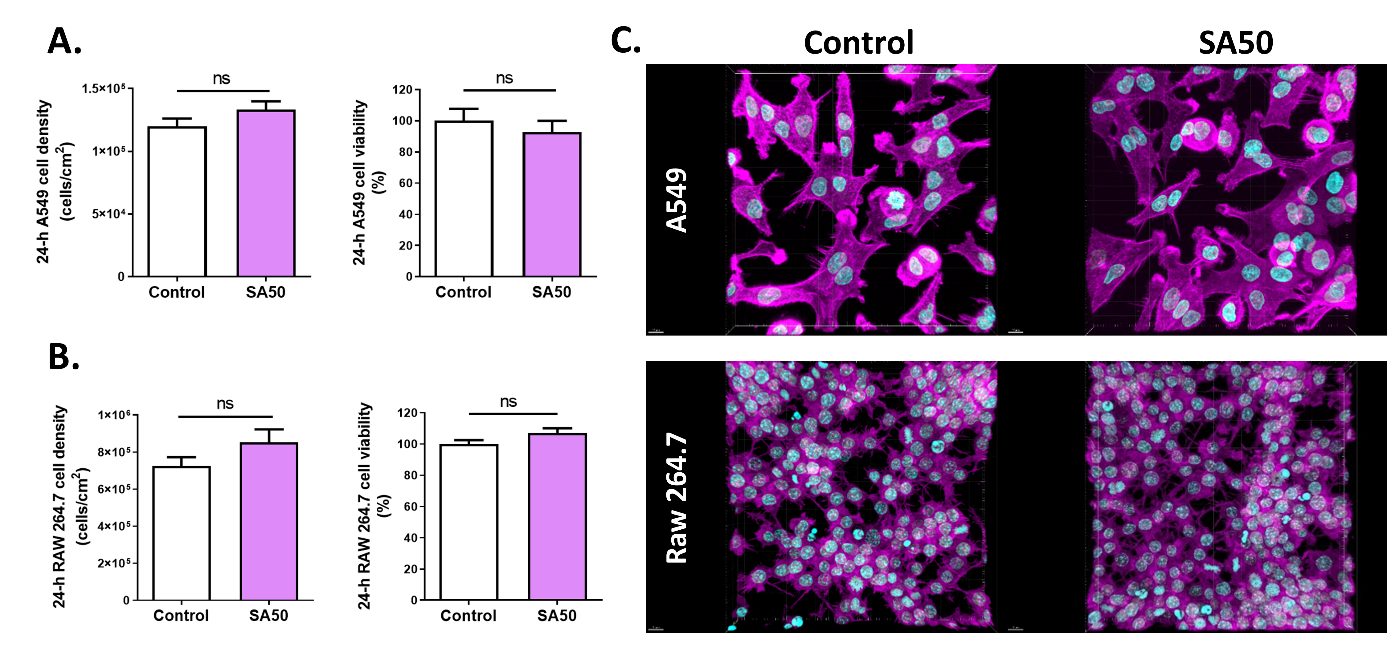


**Figure S9. Biocompatibility evaluation of unmodified PDMS and serum albumin- preadsorbed PDMS surfaces.** Cell density and viability murine RAW 264.7 macrophages (**A**) and human lung epithelial A549 cells (**B**) on unmodified PDMS (Control) and serum albumin-coated surfaces (SA50) after 24 hours of incubation. (**C**) Representative cell morphology of RAW 264.7 and A549 cells on Control and SA50 surfaces after 24 hours. Cell density and morphology after 24 hours were assessed as described in **Section 2.6**. Image acquisition was performed using a Leica TCS SP8 CFS confocal microscope. DAPI and ActinRed were excited at 405 nm and 552 nm, respectively, and fluorescence emission was collected sequentially using a hybrid detector in photon-counting mode with band-pass filters set at 450–480 nm (DAPI) and 570–620 nm (ActinRed). Image analysis and cell quantification were carried out using Imaris 9.8 software, employing the "spot fluorescent detection" function to automatically determine cell density; Cell viability was assessed using an MTT assay. After 24 hours of incubation, cells were washed twice with PBS and incubated with 100 µL of fresh medium containing MTT reagent (0.5 mg/mL) for 2 hours at 37 °C. The MTT solution was then replaced with 100 µL of DMSO to dissolve formazan crystals, and absorbance was measured at 570 nm. Cell viability (%) was calculated as the ratio of A570 from treated cells to A570 from control cells (set as 100%). Data are presented as mean ± SEM; n = 4. Scale bar: 10 µm.


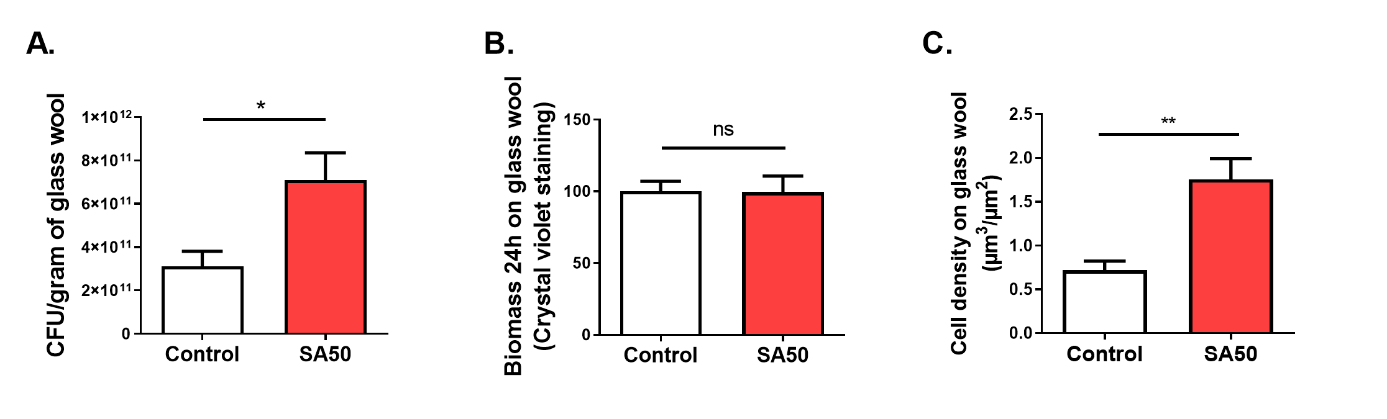


**Figure S10**. **Quantification of cell density (CFU/gram) (A), biomass (B), and biovolume (µm³/µm²) of *P. aeruginosa* adhered to unmodified glass wool fibers (Control) and SA-pre-adsorbed glass wool fibers (SA50) after 24 hours of incubation**. For cell density quantification (CFU/gram), sessile cells from *P. aeruginosa* biofilms were prepared as described in **Section 2.7.1**. After resuspension in PBS, the cells were serially diluted and plated on MH agar. Colony-forming unit (CFU) counts were normalized to the initial mass of glass wool fibers used; Biofilm biomass was quantified using the crystal violet (CV) staining method. After 24 hours of incubation, the glass wool fibers with adhered *P. aeruginosa* biofilm were washed three times with PBS and stained with CV as described in **Section 2.5**. Stained fibers were then air-dried for 24 hours to remove all residual water before solubilization in 30% acetic acid. The optical density at 580 nm of the resulting CV solution was normalized to the dry mass of the glass wool fibers prior to staining. Results are expressed as a percentage relative to the Control group; For biovolume quantification (µm^3^/µm^2^), 3D reconstructions of *P. aeruginosa* biofilms were first generated from confocal microscopy images. The total biovolume and corresponding fiber length in each image were measured. Biofilm biovolume was then normalized to the cylindrical surface area of the glass wool fiber, assuming an average diameter of approximately 20 µm. At least 10 images from 3 biological replicates were used for quantification.


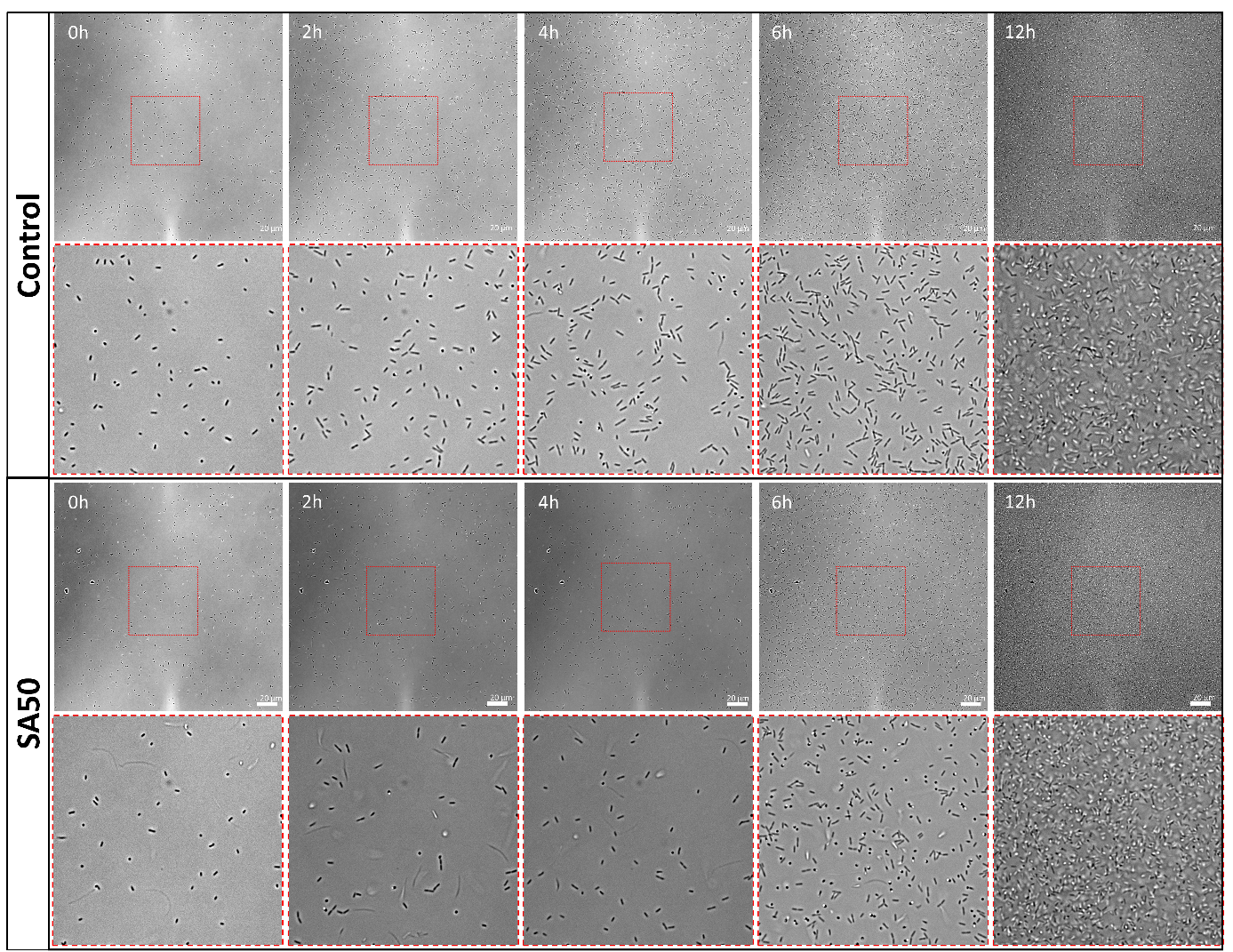


**Figure S11. Time-lapse imaging of *P. aeruginosa* adhesion on unmodified (Control) and SA-pre-adsorbed (SA50) surfaces.** This experiment was performed using MatTek glass-bottom dishes and an Olympus IX83 inverted wide-field microscope. First, the SA-pre-adsorbed surfaces were prepared as described in **Section 2.3**. Then, 3 mL of MH medium containing *P. aeruginosa* (1 × 10^7^ CFU/mL) was added to each dish. Bacterial adhesion on the MatTek dish surfaces was monitored over 24 hours, with images captured every 15 mins. *Note*: Some bacteria appear round due to the focal plane passing through the middle of the cells during imaging.


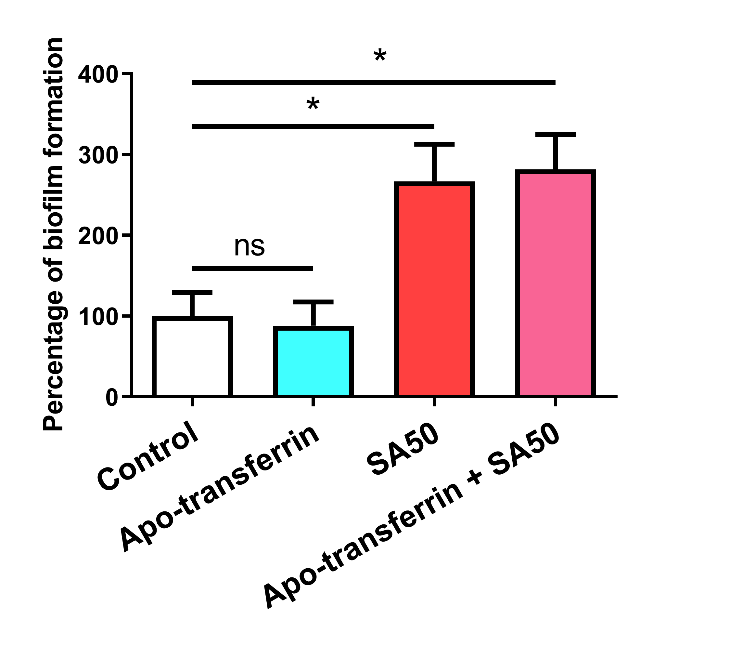


**Figure S12. Evaluation of biofilm formation on apotransferrin-preadsorbed PDMS surfaces**. Apotransferrin-preadsorbed PDMS surfaces were prepared as described in Section 2.3. Apotransferrin was used at a physiological serum-level concentration (0.1 mg/mL), either alone or in combination with SA at 50 mg/mL. *P. aeruginosa* biofilm formation was performed as described in Section 2.4. After 24 h of biofilm formation at 37 °C, bacterial biofilms were sonicated for 10 min at 45 kHz to detach cells attached to the surfaces. The resulting bacterial suspensions were plated for enumeration using the microdilution method. Results are expressed as percentages relative to the control group (unmodified surface). Data are presented as mean ± SEM; n = 6; ns: not significant, *p < 0.05.
